# Metal‐Organic Framework Glass as a Functional Filler Enables Enhanced Performance of Solid‐State Polymer Electrolytes for Lithium Metal Batteries

**DOI:** 10.1002/advs.202306698

**Published:** 2023-12-25

**Authors:** Junwei Ding, Tao Du, Emil H. Thomsen, David Andresen, Mathias R. Fischer, Anders K. Møller, Andreas R. Petersen, Andreas K. Pedersen, Lars R. Jensen, Shiwen Wang, Morten M. Smedskjaer

**Affiliations:** ^1^ Department of Chemistry and Bioscience Aalborg University Aalborg 9220 Denmark; ^2^ Department of Materials and Production Aalborg University Aalborg 9220 Denmark; ^3^ College of New Energy Zhengzhou University of Light Industry Zhengzhou 450002 China

**Keywords:** functional filler, ionic liquid, lithium metal batteries, metal‐organic framework glass, solid‐state polymer electrolyte

## Abstract

Polymers are promising candidates as solid‐state electrolytes due to their performance and processability, but fillers play a critical role in adjusting the polymer network structure and electrochemical, thermal, and mechanical properties. Most fillers studied so far are anisotropic, limiting the possibility of homogeneous ion transport. Here, applying metal‐organic framework (MOF) glass as an isotropic functional filler, solid‐state polyethylene oxide (PEO) electrolytes are prepared. Calorimetric and diffusion kinetics tests show that the MOF glass addition reduces the glass transition temperature of the polymer phase, improving the mobility of the polymer chains, and thereby facilitating lithium (Li) ion transport. By also incorporating the lithium salt and ionic liquid (IL), Li–Li symmetric cell tests of the PEO‐lithium salt‐MOF glass‐IL electrolyte reveal low overpotential, indicating low interfacial impedance. Simulations show that the isotropic structure of the MOF glass facilitates the wettability of the IL by enhancing interfacial interactions, leading to a less confined IL structure that promotes Li‐ion mobility. Finally, the obtained electrolyte is used to construct Li–lithium iron phosphate full batteries that feature high cycle stability and rate capability. This work therefore demonstrates how an isotropic functional filler can be used to enhance the electrochemical performance of solid‐state polymer electrolytes.

## Graphical abstract

A metal‐organic framework (MOF) glass is used as a functional filler to construct solid‐state polymer electrolytes. Experiments and simulations demonstrate the advantages of the isotropic and grain boundary‐free structure of the MOF glass, facilitating wettability and in turn enhanced interfacial interaction. The advanced glassy MOF can then be used to make high‐performance lithium metal batteries.

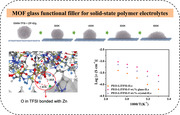

## 1 Introduction

The development of safe, high‐performance batteries is needed to enable large‐scale, efficient energy storage systems.^[^ ^1^ ^]^ Traditional lithium‐ion batteries use liquid electrolytes, but these suffer from potential safety hazards and the possibilities for increasing their energy density are limited.^[^ ^2^ ^]^ The development of lithium metal batteries based on solid electrolytes, which can simultaneously improve energy density and safety, is therefore a promising alternative.^[^ ^3^ ^]^ Among the different types of solid‐state electrolytes, polymers feature prominent properties such as processability and high stability.^[^ ^4^ ^]^ However, pure polymer electrolytes suffer from very low ionic conductivity (typically <10^−6^ S cm^−1^ at room temperature)^[^ ^5^ ^]^ and are thus not suitable for direct use in solid‐state lithium metal batteries.^[^ ^6^ ^]^

Strategies to enhance the performance of polymer solid‐state electrolytes include network structure design, quasi‐solid‐state modification, and filler modification.^[^ ^7^ ^]^ Considering the need for continuous processability in large‐scale applications,^[^ ^8^ ^]^ a promising strategy is to use cheap and easy‐to‐obtain polymers such as polyethylene oxide (PEO).^[^ ^9^ ^]^ Furthermore, filler modification can be used to simultaneously improve ionic diffusivity, mechanical properties, electrochemical properties, and thermal stability.^[^ ^10^ ^]^ The fillers reported so far are categorized as inert, active, and functional.^[^ ^11^ ^]^ Examples of inert fillers include silica, titania, and alumina, but their effect on the electrolyte performance is very limited as they cannot transfer ions.^[^ ^12^ ^]^ Active fillers are typically inorganic solid electrolytes, such as oxides, chlorides, and sulfides,^[^ ^13^ ^]^ and these feature excellent ionic conductivity. Recently, functional fillers have emerged as the most promising category due to their multiple enhancement effects. They are typically based on two dimensional (2D) materials, succinic acid, or metal‐organic frameworks (MOFs).^[^ ^11^ ^]^ MOFs are especially promising candidates due to their tunable inorganic and organic components, pore structures, and framework topology.^[^ ^14^ ^]^

Enhancing the interaction between MOF fillers and polymer networks is the key to improving the performance of polymer solid‐state electrolytes. For example, this is possible through surface chemical modification of MOFs with, for example, imidazole‐type ionic liquid (IL), thereby improving the electrochemical and mechanical properties of polymer electrolytes.^[^ ^14b^ ^]^ This is because room‐temperature ILs exhibit high ionic conductivity, high thermal stability, anti‐flammability, non‐volatility, and wide electrochemical window,^[^ ^15^ ^]^ making them promising functional plasticizers for enhancing the electrochemical performance of polymer electrolytes without sacrificing the mechanical properties.^[^ ^16^ ^]^ To our knowledge, all of the investigated MOF fillers in polymer electrolytes are crystalline.^[^ ^17^ ^]^ However, it has recently been discovered that it is possible to prepare MOF glasses, which are isotropic and feature long‐range structural disorders. Consequently, there are no grain boundaries in bulk MOF glasses and the surface of MOF glasses is uniform, unlike MOF crystals.^[^ ^18^ ^]^ In particular, zeolitic imidazolate frameworks (ZIFs) have been found to form glasses using the melt‐quenching method,^[^ ^19^ ^]^ and ZIF‐4 glass (Zn(Im)_2_, where Im is imidazolate) has been used to prepare quasi‐solid electrolytes for lithium metal batteries with remarkable performance.^[^ ^20^ ^]^

**Figure 1:**
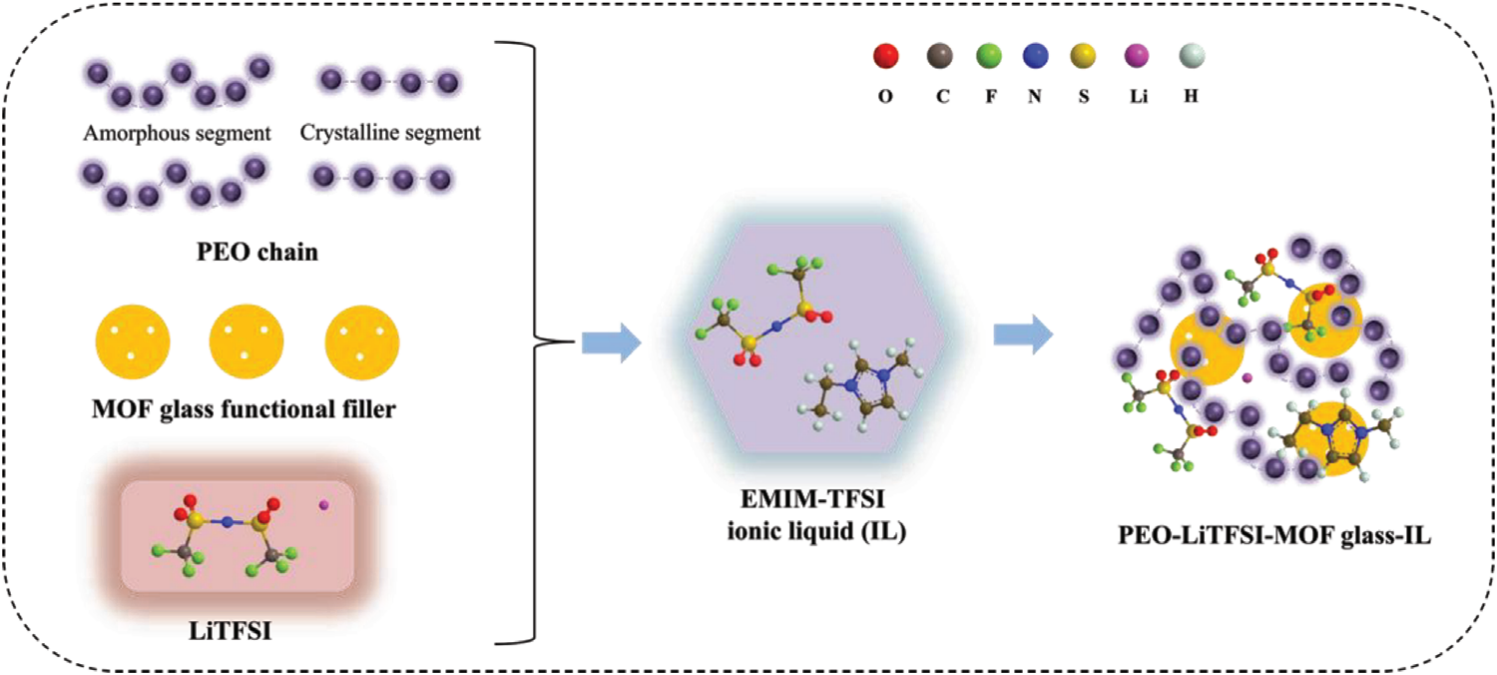
Schematic diagram of the preparation of MOF glass functional filler‐based solid‐state polymer electrolytes with ILs.

Among the different ZIFs, ZIF‐62 glass (Zn(Im)_1.75_(bIm)_0.25_, where bIm is benzimidazolate) is found to be an especially good glass‐former,^[^ ^21^ ^]^ and the presence of bulkier benzimidazoles could enable larger ion transport channels to improve the ionic diffusion capability. Indeed, ZIF‐62 glass has recently been used as an anode material in lithium‐ion batteries with high lithium storage capacity.^[^ ^22^ ^]^ Due to its organic ligand structure similar to that in polymers and ILs (e.g., 1‐ethyl‐3‐methylimidazolium bis(trifluoromethylsulfonyl)imide (EMIM‐TFSI) has superior intrinsic ionic conductivity and low viscosity), strong molecular‐level interactions with these phases can form, creating a more uniform ion diffusion network compared with traditional inorganic fillers.^[^ ^11^ ^]^ Furthermore, the isotropic structure can shorten the ion diffusion distance compared with that in the corresponding crystals. Generally, polymer solid electrolytes need to combine high ionic conductivity with low electronic conductivity to achieve fast ion transport and prevent short‐circuiting of the battery. The insulator‐like properties of ZIF‐62 glass are thus also beneficial. In this work, we therefore use ZIF‐62 glass as a functional filler to simultaneously regulate the electrochemical performance, mechanical properties, and thermal stability of solid‐state polymer electrolytes (**Figure** **1**).

In detail, we systematically vary the added amount of ZIF‐62 glass (1, 5, and 10 wt.%) in the PEO based electrolytes. PEO is chosen as polymer due to its low cost, relatively good mechanical stability, electrode compatibility, and ion‐transport facilitation.^[^ ^23^ ^]^ To improve the ionic conductivity, we also add the lithium salt LiN(CF_3_SO_2_)_2_ (lithium bis(trifluoromethylsulphonyl)imide, LiTFSI), since such salts with relatively large anions can easily dissolve and dissociate in the PEO matrix, in turn enhancing the conduction of Li cations.^[^ ^5^ ^]^ We name these samples PEO‐LiTFSI‐1 wt.% glass, PEO‐LiTFSI‐5 wt.% glass, and PEO‐LiTFSI‐10 wt.% glass. For comparison, polymer solid electrolytes with ZIF‐62 crystal and without any filler are also prepared and named PEO‐LiTFSI‐5 wt.% crystal and PEO‐LiTFSI, respectively. We study the interactions between the ZIF glass and polymer network by X‐ray diffraction (XRD), Raman spectroscopy, and differential scanning calorimetry (DSC) tests. The mechanical properties and thermodynamic stability of solid electrolytes are also analyzed. To further enhance the potential for practical applications of the resulting polymer electrolytes, an IL is introduced. The mechanism of ZIF‐62 glass‐enhanced ionic conductivity with IL is investigated by molecular dynamics (MD) simulations. Finally, half cells, symmetrical cells, and full cells are assembled to characterize electrochemical properties (ionic conductivity, electrochemical stability window, lithium ion transference number, overpotential, and cycle performance) based on the PEO‐LiTFSI‐5 wt.% glass‐IL, PEO‐LiTFSI‐5 wt.% crystal‐IL, and PEO‐LiTFSI‐IL electrolytes.

## 2 Results and Discussion

### 2.1 Structure Analysis of ZIF‐62 Crystal and Glass

**Figure 2:**
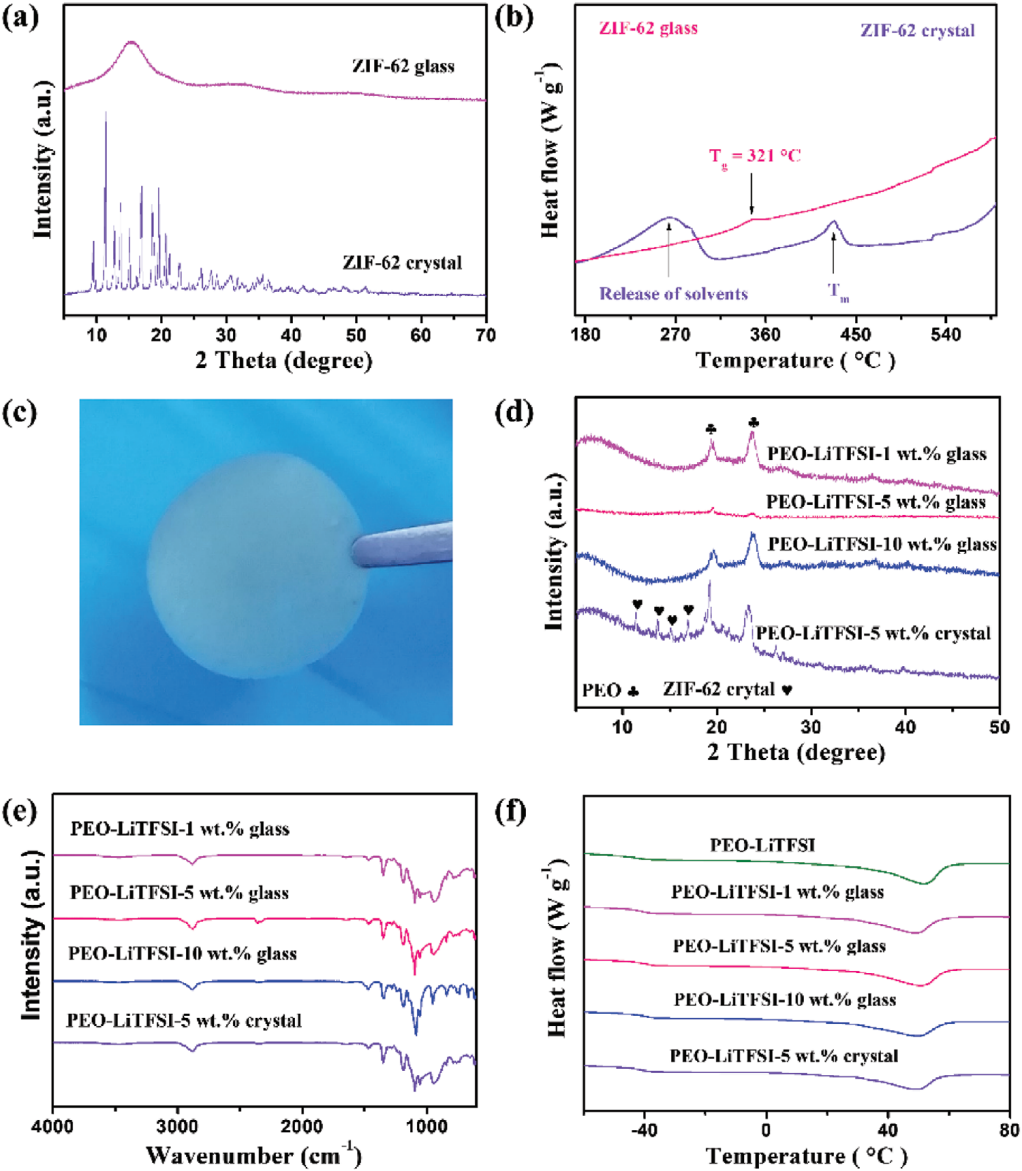
a) XRD patterns and b) DSC curves of the ZIF‐62 crystal and ZIF‐62 glass. c) Optical image of the MOF glass functional filler‐based solid‐state polymer electrolytes without IL. d) XRD patterns, e) FTIR spectra, and f) DSC curves of different electrolytes.

To confirm the structures of the prepared ZIF‐62 crystals and glasses, we first analyze the XRD results (**Figure** **2**a). The characteristic narrow peaks confirm the successful preparation of the ZIF‐62 crystal, in agreement with the literature.^[^ ^24^ ^]^ Upon melt‐quenching, the sharp peaks disappear and a new broad peak is observed due to the amorphous (disordered) nature of the glassy sample.^[^ ^25^ ^]^^1^H nuclear magnetic resonance (NMR) is further applied to determine the molecular composition of the linkers in the ZIF‐62 crystal and glass. As seen in Figure S1, Supporting Information, the benzimidazole/imidazole ratio of ZIF‐62 crystal and glass are 0.26/1.74 (ZnIm_1.74_bIm_0.26_) and 0.23/1.77 (ZnIm_1.77_bIm_0.23_), respectively. These NMR results confirm that the composition is stable during the melt‐quenching process, as found previously for the chosen melting temperature and durations.^[^ ^25^ ^]^ To further compare the structures of the ZIF‐62 crystal and glass, we show the Fourier‐transform infrared (FTIR) spectroscopy in Figure S2, Supporting Information. All peaks below 1200 cm^−1^ are attributed to C─H bonds in the benzimidazolate and imidazolate ring structures. Among them, all peaks between 1200 and 1000 cm^−1^ are caused by the C─H in‐plane vibration, and the peaks below 1000 cm^−1^ are caused by the C─H out‐of‐plane vibration. The peaks between 1600 and 1200 cm^−1^ can be attributed to C═C/C─C, C─N, and C═N bonds in the aromatic structure of benzimidazolate and imidazolate units.^[^ ^26^ ^]^ Only a few differences between the ZIF‐62 glass and crystal can be observed, including the peak around 910 cm^−1^, which is found for crystal ZIF‐62 but not for ZIF‐62 glass. Overall, the FTIR results confirm that the short‐range chemical bonding structures in the ZIF‐62 glass are similar to those in the ZIF‐62 crystal. The DSC results presented in Figure 2b are used to compare the calorimetric information of the ZIF‐62 crystal and glass. We observe an endothermic peak upon heating of the ZIF‐62 crystal at ≈250 °C due to the evaporation of the solvent DMF in ZIF‐62 crystal micropores.^[^ ^27^ ^]^ The melting temperature of the ZIF‐62 crystals can be determined as the endothermic peak between 400 and 450 °C, while the glass transition temperature (*T*_g_) of ZIF‐62 glass is identified at 321 °C. These values match well with those reported in the literature.^[^ ^27^ ^]^ Meanwhile, compared with ZIF‐62 crystals, the ZIF‐62 glass obtained through the melt‐quenching process has a smaller particle size (Figure S3, Supporting Information).

### 2.2 Characterization of Electrolyte Films with and without IL

We first analyze the structures of the obtained electrolyte films via the solvent‐casting method (Figure 2c) by considering the XRD results (Figure 2d and Figure S4, Supporting Information). No obvious LiTFSI‐related peaks are observed in any of the obtained electrolyte films, which indicates that LiTFSI is completely dissolved in PEO and thereby not in a crystalline state. The addition of ZIF‐62 glass or crystal does not affect the dissolution of LiTFSI in the PEO network. All films display two peaks at 19° and 23°, which can be ascribed to the crystalline PEO structure. Since PEO itself is partly crystalline and amorphous and the added amount of ZIF‐62 glass is relatively small, XRD cannot detect the broad peak corresponding to ZIF‐62 glass. However, the sharp peaks due to ZIF‐62 crystal can be identified in the PEO‐LiTFSI‐5 wt.% crystal electrolyte film, indicating that the ZIF‐62 crystals are not (fully) dissolved in the PEO network. To further analyze the vibrational bands of the prepared electrolyte films, we consider the FTIR test results (Figure 2e and Figure S5, Supporting Information). The peaks due to the PEO, LiTFSI, and the ZIF‐62 glass/crystal components are found in all the films, which confirms a mixture of components has been successfully synthesized.^[^ ^28^ ^]^ Importantly, the peak observed at around 1354 cm^−1^ is ascribed to the asymmetric S═O stretching of TFSI^−^ (the S═O of its own structure is a symmetrical vibration mode), which confirms the interaction between PEO and LiTFSI.^[^ ^14b^ ^]^

We next determine the *T*_g_ of PEO in the fabricated electrolyte films through calorimetric analysis in the temperature range from −60 to 100 °C (Figure 2f). The values of *T*_g_ give insight into which films have the most mobile PEO segments and therefore potentially show the best electrochemical performance.^[^ ^14c^ ^]^ For pure PEO, *T*_g_ is −45.3 °C, while the electrolyte films with 1 wt.% glass, 5 wt.% glass, 10 wt.% glass, and 5 wt.% crystal exhibit *T*_g_ values of −45.6, −45.8, −45.7, and −45.6 °C, respectively. That is, the addition of MOF glass/crystal functional filler slightly lowers the polymer *T*_g_. This is likely because MOF glass/crystal as the plasticizer can reduce the regular arrangement of chain segments and thus decrease the *T*_g_ of the PEO‐LiTFSI matrix, which is consistent with previous results for an MOF crystal.^[^ ^14c^ ^]^ Based on DSC results,^[^ ^14c^ ^]^ the calculated degree of crystallinity for PEO‐LiTFSI, PEO‐LiTFSI‐1 wt.% glass, PEO‐LiTFSI‐5 wt.% glass, PEO‐LiTFSI‐10 wt.% glass, and PEO‐LiTFSI‐5 wt.% crystal electrolyte films are 16.2%, 14.1%, 13.6%, 13.7%, and 13.9%, respectively. Low crystallinity is beneficial to achieve high ionic conductivity. To confirm that the ZIF‐62 glass and crystal are dispersed homogenously in the PEO and LiTFSI systems, we consider the optical microscope analyses (Figure S6a–e, Supporting Information). The images show that the surfaces of the films change as a function of the ZIF‐62 content. At low concentrations, the surface mainly consists of small spheres of ≈40 µm in diameter. These spheres have previously been ascribed to crystalline PEO.^[^ ^29^ ^]^ If the concentration is too high, there will be agglomeration of the functional fillers. At the concentration of 5 wt.% ZIF‐62 glass, the surface appears more smooth in comparison to that of 5 wt.% ZIF‐62 crystal, indicating that the electrolyte film with glass phase is homogeneously prepared. To explore the mechanical behavior of the electrolyte films, microindentation experiments have been performed (Figure S6f, Supporting Information). We determine the micro‐hardness by analyzing the unloading displacement curves (Figures S7 and S8 and Table S1, Supporting Information), finding that the prepared electrolyte films feature similar hardness. Furthermore, stretching of the films reveals that the PEO‐LiTFSI‐5 wt.% glass electrolyte can be elongated to the largest extent among the samples (Figure S6f, Supporting Information).

**Figure 3:**
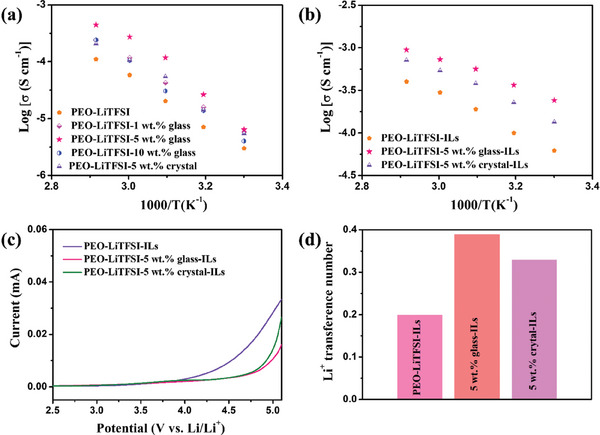
a,b) Temperature dependence of ionic conductivity of the different solid‐state polymer electrolytes without (a) and with (b) EMIM‐TFSI IL. c) Linear sweep voltammograms of solid‐state polymer electrolytes with EMIM‐TFSI IL at 60 °C. d) Lithium‐ion transference number of solid‐state polymer electrolytes with EMIM‐TFSI IL.

Next, we determine the ionic conductivity (*σ*) of the electrolyte films (**Figure** **3**a and Figures S9 and S10, Supporting Information). As shown in previous work, the ionic conductivity at room temperature of pure PEO‐LiTFSI is around 10^−6^ S cm^−1^.^[^ ^30^ ^]^ As expected based on the polymer segment regulation functionality of fillers, the addition of ZIF‐62 glass and crystal to PEO‐LiTFSI hinders the crystallization of PEO and thus improves the ionic conductivity. The highest ionic conductivity at 50 °C (7.13 × 10^−5^ S cm^−1^) is obtained for the electrolyte containing 5 wt.% ZIF‐62 glass. Therefore, we choose the PEO‐LiTFSI‐5 wt.% glass electrolyte sample for further analysis and compare it with the PEO‐LiTFSI and PEO‐LiTFSI‐5 wt.% crystal electrolytes. These results suggest that the polymer solid‐state electrolytes based only on PEO, ZIF‐62 glass/crystal, and LiTFSI are insufficient for an efficient battery even at 60 °C due to their low ionic conductivity. Therefore, to significantly increase the ionic conductivity of the electrolyte, we have added a small amount of IL (namely, EMIM‐TFSI) with superior intrinsic ionic conductivity, low viscosity, and melting point (−12 °C), high thermal stability (degradation temperature above 400 °C), and high chemical stability.^[^ ^31^ ^]^ Upon the addition of 5 µL IL, the ionic conductivity increases by about one order of magnitude (Figure 3b). Again, the PEO‐LiTFSI‐5 wt.% glass‐IL electrolyte exhibits the highest ionic conductivity in comparison to PEO‐LiTFSI‐5 wt.% crystal‐IL and PEO‐LiTFSI‐IL electrolytes. Furthermore, the ionic conductivity of the PEO‐LiTFSI‐5 wt.% glass‐IL electrolyte increases from 2.41 × 10^−4^ at 30 °C to 9.46 × 10^−4^ S cm^−1^ at 70 °C. Compared to the samples with ZIF‐62 crystal functional filler (1.35 × 10^−4^ S cm^−1^ at 30 °C) and no filler (6.20 × 10^−5^ S cm^−1^ at 30 °C), adding the ZIF‐62 glass gives rise to the largest increase in ionic conductivity upon IL addition. This is likely due to the isotropic nature of the ZIF‐62 glass, providing a stronger synergistic effect with the IL.

The electrochemical stability window is evaluated by linear sweep voltammetry (LSV) (Figure 3c). The stable voltages of both the PEO‐LiTFSI‐5 wt.% glass‐IL and PEO‐LiTFSI‐5 wt.% crystal‐IL electrolytes show significant improvement compared to the PEO‐LiTFSI‐IL electrolyte (≈4 V). In addition, the PEO‐LiTFSI‐5 wt.% glass‐IL electrolyte delivers a wider steady voltage (≈4.7 V) than the PEO‐LiTFSI‐5 wt.% crystal‐IL electrolyte (≈4.5 V), implying improved electrochemical stability. This improvement when using both the ZIF‐62 glass and crystal as functional fillers compared with PEO‐LiTFSI‐IL electrolyte is due to the strong interaction between the ZIF‐62 glass/crystal and PEO, which protects the ether functional groups on the PEO chains.^[^ ^32^ ^]^ The isotropic nature of the ZIF‐62 glass in PEO appears to provide a more efficient interaction with PEO, resulting in the better electrochemical stability of PEO‐LiTFSI‐5 wt.% glass‐IL relative to that of PEO‐LiTFSI‐5 wt.% crystal‐IL. The lithium‐ion transference number (*t*_Li_) is then obtained by chronoamperometry and electrochemical impedance spectroscopy (EIS) measurements (Figure 3d and Figure S11, Supporting Information). The PEO‐LiTFSI‐5 wt.% glass‐IL electrolyte delivers the highest *t*_Li_ of 0.39 relative to those of PEO‐LiTFSI‐5 wt.% crystal‐IL (0.33) and PEO‐LiTFSI‐IL (0.20) electrolytes.

### 2.3 MD Simulations

We expect three types of bulk‐phase lithium‐ion transport mechanisms to be present for the obtained three‐component ZIF‐62 glass/crystal, IL, and PEO systems. Obviously, the lithium‐ion transport in the IL is the fastest, as the ionic conductivity of EMIM‐TFSI is 8.8 × 10^−3^ S cm^−1^ at room temperature.^[^ ^31^ ^]^ PEO has moderate lithium ion conductivity (≈2.6 × 10^−5^ S cm^−1^ at 70 °C).^[^ ^33^ ^]^ Bulk ZIF‐62 glass has higher ionic conductivities than those of ZIF‐62 crystal,^[^ ^20^ ^]^ although both values are lower than that of PEO. Traditional porous materials usually confine the IL inside its pores, which increases the ion diffusion distance. According to the pore size of ZIF‐62 glass/crystal (<0.5 nm) and the size of the applied IL (EMIM‐TFSI, ≈0.8 nm), the IL cannot enter the pore structure of the ZIF‐62 phases.^[^ ^34^ ^]^ Therefore, this IL should tend to form a nanometer‐thick surface modification layer on the surface of ZIF‐62 glass and crystal particles. Furthermore, since the length of the TFSI^−^ anion (with length and width of 0.79 and 0.29 nm, respectively)^[^ ^35^ ^]^ for the lithium salt (LiTFSI) is larger than the pore sizes in the ZIF‐62 crystal and glass, most of the TFSI^−^ anions cannot enter the interior of the ZIF‐62 structures and can only reside on their surface. Given that the weight fraction of the ZIF‐62 glass/crystal and IL in electrolytes is relatively low, most of the lithium ions are still transported through the PEO network, and only a small amount of lithium ions transport on the surface of ZIF‐62 glass/crystal particles. That is, we expect ZIF‐62 glass and crystal to interact with the IL and LiTFSI, and therefore, this interaction should play a major role in improving the dissociation of IL and LiTFSI and then enhance the ionic conductivity.

**Figure 4:**
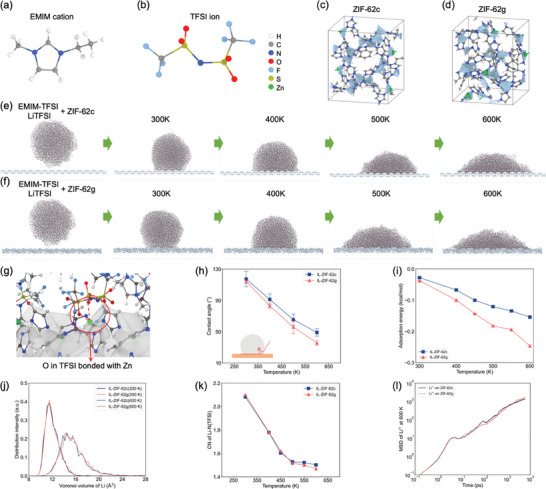
Schematic of the atomic structure of a) EMIM cation, b) TFSI ion, c) ZIF‐62 crystal, and d) ZIF‐62 glass. Snapshot of the wetting behavior simulations of LiTFSI/EMIM‐TFSI IL on the surface of e) ZIF‐62 crystal and f) ZIF‐62 glass after equilibrating at different temperatures for 6 ns. g) Interfacial structure between IL and ZIF‐62 crystal, where the ZIF phase is wrapped with a grey shading for better visualization. h) Contact angle of an IL droplet on different ZIF‐62 substrates as a function of temperature. i) Absorption energy of ILs on the surface of different ZIF‐62 substrates as a function of temperature. Note that the energy is normalized by the number of atoms within the IL. j) Atomic volume of Li^+^ within ILs on different ZIF‐62 substrates at different temperatures. k) Number of N atoms in TFSI^−^ coordinated to Li^+^ within a cutoff of 4 Å as a function of temperature. j) MSD curves of Li^+^ in IL different ZIF‐62 substrates at 600 K.

To understand this effect, MD simulations have been carried out to understand the wettability of the IL on the surface of the ZIF‐62 phases and clarify how the surface confinement on IL influences the subsequent diffusion of Li^+^ within the IL. As shown in **Figure** **4**a,b, we first build the IL model by mixing EMIM and Li cations with TFSI ions to obtain a homogeneous droplet. The crystalline and glassy ZIF‐62 structures are shown in Figures 4c,d, respectively, highlighting the Zn‐centered tetrahedra and pore structure surrounded by imidazole linkers. When the LiTFSI/EMIM‐TFSI droplet is placed near the surface of the ZIF‐62 substrates, it spreads over the surface due to the interatomic interactions. With an increase in temperature, the initially spherical droplet gradually changes to an arc‐shaped configuration, indicating a decrease in the contact angle (Figure 4e,f). Interestingly, the LiTFSI/EMIM‐TFSI droplet on the glassy surface tends to exhibit lower height than that on the crystalline surface, suggesting that the surface of ZIF‐62 glass has a stronger affinity for the LiTFSI/EMIM‐TFSI phase. The interfaces feature many defective Zn atoms (i.e., Zn atoms with coordination number below 4) between IL and ZIF‐62 crystal/glass (Figure 4g), and the defective Zn atom can be coordinated with the O atoms in the TFSI ions through Coulombic interactions. Since the surface of ZIF‐62 glass is flatter than that of the crystalline phase (Figure S12, Supporting Information), the exposed defective Zn atoms are more uniformly distributed on the surface to facilitate ionic bonding. We further quantify the interaction between the IL and ZIF substrates by calculating the contact angle and adsorption energy (Figure 4h,i). As expected, both the contact angle and adsorption energy decrease as the temperature increases, reflecting the increase in the contact area.

The evolution of the adsorption energy of IL on different ZIF‐62 surfaces during the equilibration process is shown in Figure S13, Supporting Information. The IL exhibits a better wettability on the glass compared to the crystalline surface of ZIF‐62 due to the stronger interactions. The atomic volume of Li^+^ within the IL is also influenced by the surface confinement of ZIF‐62 substrates as the atomic volume of Li^+^ on ZIF‐62 glass is generally larger than that on ZIF‐62 crystal due to the lower surface confinement of ZIF‐62 glass (Figure 4j). The dissolution of LiTFSI on ZIF‐62 glass is generally faster than that on ZIF‐62 crystal, as manifested by the lower Li–N coordination (Figure 4k). The mean squared displacement (MSD) of Li^+^ on ZIF‐62 glass is generally slightly higher than that on ZIF‐62 crystal (Figure 4l and Figure S14, Supporting Information), thus confirming the relatively higher ionic conduction of Li^+^ on ZIF‐62 glass. Therefore, via both lowering the surface confinement of IL and promoting the dissolution of LiTFSI, the Li^+^ conductivity can be improved. Based on the Young–Laplace equation,^[^ ^36^ ^]^ the larger contact angle induces a larger internal pressure within the liquid.^[^ ^37^ ^]^

**Figure 5:**
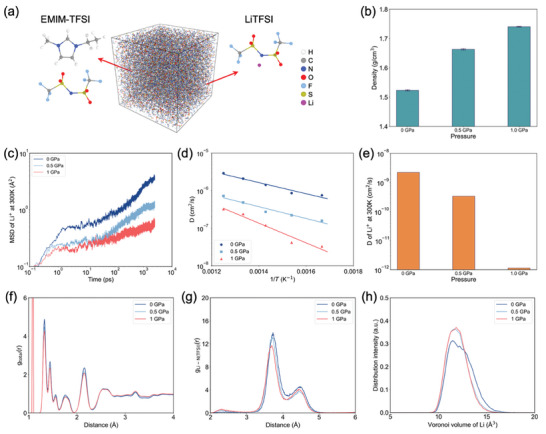
a) Schematic of the simulation model of ILs with Li^+^. b) Density of the simulated model under different pressures. c) MSD curves of Li^+^ in IL at 300 K for different pressures. d) Temperature dependence of the diffusion coefficient of Li^+^ at the different pressures. Lines represent fits to the Arrhenius equation. e) Diffusion coefficient of Li^+^ at 300 K calculated from the Arrhenius correlation for the different pressures. f) Total and g) Li–N partial pair distribution function of IL under different pressures. h) Distribution of atomic volume of Li^+^ in different IL calculated from Voronoi tessellation for different pressures.

In the following, we therefore further investigate the diffusion behavior of Li ions in ILs under different pressures as the surface confinement of ZIF‐62 leads to the changes in the atomic volume of Li^+^. Surface confinement of ILs can lead to a capillary pressure of up to a hundred MPa,^[^ ^38^ ^]^ we select pressures of 0, 0.5, and 1 GPa to magnify the effect, as it is usually done in MD simulations.^[^ ^39^ ^]^ The initial configuration of EMIM‐TFSI and Li‐TFSI molecules is shown in **Figure** **5**a. After being equilibrated at 300 K, the density of the system increases with an increase in the applied pressure (Figure 5b). As shown in Figure 5c, the MSD curve of Li ions decreases with increasing pressure, indicating that the mobility of the ions is reduced at high pressure. Since the MSD curves did not reach the diffusive regime (as manifested by a slope of 1 in the log‐log plot), we cannot directly use the Einstein relation (Experimental Section, Equation (3)) to calculate the diffusion coefficient. Instead, we simulate the MSD curves at elevated temperatures (Figure S15, Supporting Information), finding that the mobility of ions is enhanced at elevated temperatures, while the mobility is reduced by the applied pressure regardless of the temperature. We then calculate the diffusion coefficient at 300 K by relying on the Arrhenius temperature dependence of diffusion (Figure 5d). The calculated diffusion coefficient at 300 K is shown in Figure 5e, showing reduced mobility by pressure, which also agrees with literature results on the IL of 1‐butyl‐3‐methylimidazolium hexafluorophosphate ([C_4_mim]‐[PF_6_]).^[^ ^39^ ^]^

The mechanism of the reduced mobility is further analyzed by calculating the pair distribution function (PDF) and the atomic volume of Li. The total PDF shown in Figure 5f reveals that the applied pressure does not induce an obvious change in the bonding structure of the atomic pairs. The partial PDF of the Li–N pair shown in Figure 5g indicates that the distance between Li^+^ and TFSI ion decreases under high pressure, which leads to reduced space for Li^+^, in turn hindering Li^+^ movement (Figure 5h). Based on the simulation results, we conclude that the highly uniform structure of ZIF‐62 glass enables the exposure of defective Zn sites, which in turn gives rise to a stronger interaction with the ions of the IL to ensure better wettability. This stronger interfacial interaction leads to a less confined environment for the IL to facilitate ionic conduction. Combined with the existing dissociation mechanism of IL and LiTFSI in MOF‐based polymer electrolytes,^[^ ^40^ ^]^ the isotropic surface of ZIF‐62 glass particles cannot only achieve a more uniform IL distribution than that of ZIF‐62 crystal but also facilitate the uniform interaction as well as the IL and LiTFSI dissociation. Both of these effects can improve ion transport in the PEO‐IL solid‐state electrolyte system.

### 2.4 Electrochemical Properties and Performances

**Figure 6:**
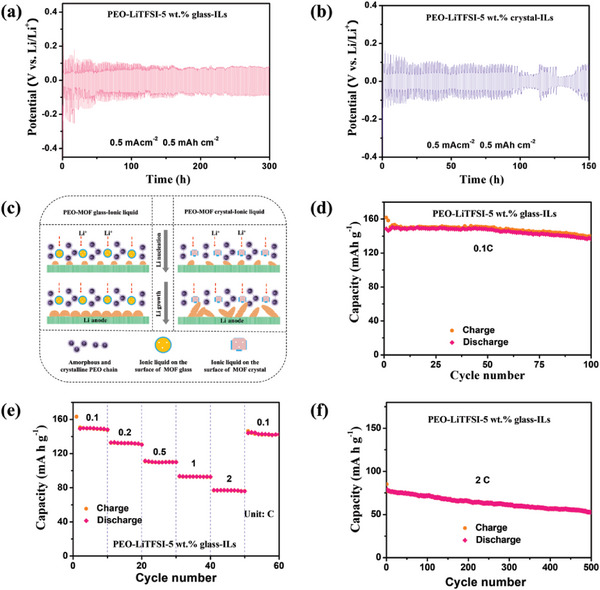
a,b) Voltage evolution curves at 60 °C of a) PEO‐LiTFSI‐5 wt.% glass‐IL and b) PEO‐LiTFSI‐5 wt.% crystal‐IL electrolytes based on Li–Li symmetric batteries. c) Schematic of Li nucleation and growth processes for PEO‐LiTFSI‐5 wt.% glass‐IL and PEO‐LiTFSI‐5 wt.% crystal‐IL electrolytes. d) Cyclic, e) rate, and f) long cycle performance of the PEO‐LiTFSI‐5 wt.% glass‐IL electrolyte‐based Li‐LFP full batteries at 60 °C.

Given the promising results above, we further explore the lithium plating/stripping performance by assembling and testing Li|electrolyte|Li symmetrical batteries (**Figures** **6**a,b and S16, Supporting Information). The average overpotential for the PEO‐LiTFSI‐5 wt.% glass‐IL electrolyte is about 75 mV, which is lower than those of PEO‐LiTFSI‐IL (≈96 mV) and PEO‐LiTFSI‐5 wt.% crystal‐IL electrolytes (≈91 mV). Furthermore, the PEO‐LiTFSI‐5 wt.% glass‐IL electrolyte can function for 300 h, which is longer than the other two electrolytes, as PEO‐LiTFSI‐5 wt.% crystal‐IL and PEO‐LiTFSI‐IL can only function for ≈100 h and ≈75 h before short circuit, respectively. The long cycle stability for the glass‐based filler indicates uniform lithium plating and stripping processes in this sample (Figure 6c).

A comparison of these performances with the state‐of‐the‐art solid‐state electrolytes from the literature shows that the PEO‐LiTFSI‐5 wt.% glass‐IL electrolyte is a good candidate material (Table S2, Supporting Information). To then test the full battery performance, we assemble Li|electrolyte|lithium iron phosphate (LFP) batteries. As shown in Figure 6d and Figures S17–S19, Supporting Information, the PEO‐LiTFSI‐5 wt.% glass‐IL electrolyte‐based full‐battery exhibits higher capacity (≈148 mAh g^−1^) compared with those of PEO‐LiTFSI‐5 wt.% crystal‐IL (≈134 mAh g^−1^) and PEO‐LiTFSI‐IL (≈116 mAh g^−1^) electrolytes, indicating that the addition of the ZIF‐62 glass facilitates the Li‐ion transfer. The cyclability at different current densities is then explored by performing rate performance tests. The full battery with the PEO‐LiTFSI‐5 wt.% glass‐IL electrolyte delivers capacities of about 133, 110, 92, and 76 mAh g^−1^ at 0.2, 0.5, 1 and 2 C rates, respectively (Figure 6e and Figure S20, Supporting Information). For full batteries with PEO‐LiTFSI‐5 wt.% crystal‐IL and PEO‐LiTFSI‐IL electrolytes, the battery capacities are lower and we observe significant voltage polarization when cycled at high current densities (Figures S21 and S22, Supporting Information). Compared with the other two electrolytes, PEO‐LiTFSI‐5 wt.% glass‐IL electrolyte achieves small polarization and high capacity in full batteries during high‐rate cycling, which is likely due to its higher ionic conductivity and flexibility. These results reveal that the structure of the PEO‐LiTFSI‐5 wt.% glass‐IL electrolyte is stable when cycling at a high current and also further confirm that the PEO‐LiTFSI‐5 wt.% glass‐IL electrolyte exhibits good interfacial stability on both cathode and lithium metal anode sides even during high charging/discharging rates. Furthermore, the full battery with the PEO‐LiTFSI‐5 wt.% glass‐ILs electrolyte has good long cyclic performance (Figure 6f). The full battery with the PEO‐LiTFSI‐5 wt.% glass‐ILs electrolyte can also work at room temperature (Figure S23, Supporting Information).

Based on the above electrochemical analyses, we conclude that the prepared PEO‐LiTFSI‐5 wt.% glass‐IL electrolyte features superior comprehensive properties such as high ionic conductivity/diffusion number, large voltage window, good interface stability with lithium anode, and good compatibility with cathode. Both good lithium metal anode and cathode interface stability behaviors are consistent with previous reports.^[^ ^14^, ^20^, ^40^ ^]^ Compared with ZIF‐62 crystal as the functional filler, the use of a glass phase with its isotropic particle surface improves the comprehensive performance of the polymer electrolyte. Therefore, following the design ideas of glassy functional fillers, we expect advanced polymer solid‐state electrolytes can be developed in the future. For example, the particle size and internal pore size of MOF glasses can be optimized to expose more surface and internal sites. In addition, the type and structure of the organic ligands can be adjusted, for example through post‐synthetic modification,^[^ ^41^ ^]^ to further adjust their structural match with the polymer network, IL, and poly (ionic liquid). This could enable the development of new functional solid‐state electrolytes with asymmetric structure, concentration‐gradient structure, or phase‐separation structure.

## 3 Conclusion

We have prepared a novel PEO‐based solid‐state electrolyte using a ZIF‐62 glass as a functional filler. The PEO‐LiTFSI‐5 wt.% glass electrolyte shows uniform distribution of the functional filler and improvements in both ionic conductivity and mechanical properties. Upon addition of an IL, the obtained PEO‐LiTFSI‐5 wt.% glass‐IL electrolyte delivers higher ionic conductivity, larger electrochemical stability window, higher lithium‐ion transference number, and better inhibition of lithium dendrites growth compared to PEO‐LiTFSI‐IL and PEO‐LiTFSI‐5 wt.% crystal‐IL electrolytes. Moreover, full batteries based on the PEO‐LiTFSI‐5 wt.% glass‐IL electrolyte exhibit superior rate capability and cycle performance. Atomistic simulations demonstrate that the superior performance of this new composite electrolyte is attributed to the uniformly distributed defects on the surface of the ZIF‐62 glass, giving rise to stronger interactions with the IL, which in turn leads to the formation of a less confined environment that facilitates ionic conductivity. This work thus provides a promising design strategy for future work through the use of nanoporous, isotropic MOF glasses to improve the electrochemical performances of polymer solid‐state electrolytes.

## 4 Experimental Section

### Chemicals

For the synthesis of the zinc‐based ZIF‐62 crystal, zinc nitrate hexahydrate (≥99%), imidazole (99.5%), and benzimidazole (98%) were acquired from Sigma‐Aldrich and dimethylformamide (DMF) (99.9%) was purchased from VWR chemicals. For the synthesis of polymer electrolyte films, PEO (M_w_ = 6 × 10^5^), EMIM‐TFSI, and LiTFSI (≥99%) were obtained from Sigma‐Aldrich, while acetonitrile (≥99.99%) was obtained from VWR chemicals. For the NMR analyses, 35% deuterium chloride (DCl) in D_2_O/DMSO‐d6 was obtained from VWR chemicals. All the chemicals were used as purchased without further purification.

### Preparation of ZIF‐62 Crystal

ZIF‐62 crystals were synthesized by sequentially adding 1.75 g zinc nitrate hexahydrate, 5.33 g imidazole, and 1.63 g benzimidazole into a beaker, followed by the addition of 50 mL DMF. The solutions were stirred for 30 min on a magnetic stirrer (≈600 rpm) before being transferred into 100 mL Teflon‐lined autoclaves. After transferring the solutions, the autoclaves were tightly sealed and placed in an oven at ambient temperature. The oven was heated to 130 °C and held at this temperature for 96 h. It was then turned off and the autoclaves were left to cool down to ambient temperature inside the oven. The synthesized crystals were recovered by decanting the crystal‐containing solution into 50 mL centrifuge beakers before rinsing the autoclaves with ≈40 mL DMF to ensure that all the crystals were recovered from the autoclaves. DMF from the autoclaves was then transferred to the centrifuge beakers. Afterward, the crystal‐containing solution was centrifuged at 4500 rpm for 3 min. After centrifugation, the excess DMF was removed, and this process was repeated four times. The dispersion of the recovered crystals was finally placed in an oven at 120 °C to dry for 72 h. The obtained ZIF‐62 crystals were ground into fine particles using an agate mortar.

### Preparation of ZIF‐62 Glass

The ZIF‐62 glass was obtained through melt‐quenching of the ZIF‐62 crystal. First, the ZIF‐62 crystals were placed in an alumina (Al_2_O_3_) crucible in the middle of an inert atmosphere furnace. Argon was used as the inert gas and a continuous flow of gas was supplied to the furnace. The furnace was allowed to be filled with argon for 30 min to ensure an inert atmosphere before heating. The ZIF‐62 crystals were heated to 320 °C at a heating rate of 10 °C min^−1^ and then the temperature was held constant for 50 min. This was done to vaporize the residual DMF that was trapped in the porous cavities of the ZIF‐62 structure prior to the melting of the crystals. Afterward, the temperature was raised to 460 °C, again at a heating rate of 10 °C min^−1^, and the temperature was held constant for 5 min. The furnace was then allowed to cool off at a rate of ≈10 °C min^−1^ until room temperature was reached. After the furnace had cooled down, and the crucible was collected, the ZIF‐62 glass was ground into fine particles using an agate mortar, and the final product was obtained as a white powder.

### Preparation of Polymer Electrolyte Films

First, 0.87 g LiTFSI was weighed and transferred to a small Erlenmeyer flask. The appropriate amount of ZIF‐62 glass or crystal was weighed, corresponding to a weight ratio of 0 wt.% (pure PEO‐LiTFSI), 1 wt.% (i.e., 0.03 g), 5 wt.% (i.e., 0.15 g), and 10 wt.% (i.e., 0.32 g) of the total weight of the dry reagents, and added to the Erlenmeyer flask. Afterward, 40 mL of acetonitrile was measured using a measuring cylinder and transferred to the Erlenmeyer flask. The solution was sealed tightly and placed on a magnetic stirrer and stirred for 1 h at 60 °C. To ensure a homogenous solution, it was ultrasonicated in a Branson 3210 ultrasonic bath for 30 min. The solution was then placed on a magnetic stirrer and stirred at 60 °C for 30 min, after which the heating was turned off. Then, 2 g PEO was weighed and slowly poured into the solution to obtain an EO:LiTFSI ratio of 15:1. This solution was stirred for about 18 h at ambient temperature without being sealed, allowing the acetonitrile to evaporate naturally. Afterward, the solution was heated to 60 °C for ≈3 h. Then the heating was turned off, the solution was sealed and the stirring remained on until casting. The films were cast onto different Teflon materials with a diameter of ≈50 mm by a drop‐casting method. A spoon was filled with the solution, corresponding to an amount of ≈2.5 mL, and poured onto the substrates, yielding thicknesses of the undried films of ≈1.3 mm.

The cast films were dried in a fume hood for 6 h and then in a vacuum oven at 45 °C for 20 h. After drying, the films were carefully peeled off the Teflon substrates and cut into circular electrolyte films with a diameter of 19 mm with a TMAX‐CP60 Disc Punching Machine. The MOF volume fraction values of PEO‐LiTFSI‐1 wt.% glass, PEO‐LiTFSI‐5 wt.% glass, PEO‐LiTFSI‐10 wt.% glass, and PEO‐LiTFSI‐5 wt.% crystal electrolyte films were 0.82%, 4.00%, 8.12%, and 4.19%, respectively. The thicknesses of the electrolyte films were measured using a Mitutoyo Quantamike 293 micrometer by sandwiching the films between two pieces of stainless steel (SS). To further prepare the PEO‐LiTFSI‐ILs, PEO‐LiTFSI‐5 wt.% glass‐ILs, and PEO‐LiTFSI‐5 wt.% crystal‐ILs electrolyte samples, 5 µL ionic liquid of EMIM‐TFSI was added onto the obtained 19 mm circular electrolyte films. The theoretical MOF volume fraction values of PEO‐LiTFSI‐5 wt.% glass‐ILs (3.95%) and PEO‐LiTFSI‐5 wt.% crystal‐ILs (4.15%) electrolyte samples were also different. The theoretical EMIM‐TFSI IL volume fraction values of PEO‐LiTFSI‐5 wt.% glass‐ILs and PEO‐LiTFSI‐5 wt.% crystal‐ILs electrolyte samples were 1.030% and 1.028%, respectively. However, it was noted that when transferring the solution of LITFSI, ZIF‐62 glass/crystal, and PEO in acetonitrile from the container to the PTFE substrate, it was inevitable that a small amount of solution could not be transferred from the container. When using the disc punching machine to cut the dried electrolyte films into circular electrolyte films, there will be an additional loss of the electrolyte films. Since the loss of electrolyte films cannot be effectively calculated, the IL content was calculated based on the total mass of the original amount of LITFSI, ZIF‐62 glass/crystal, and PEO. In view of the loss of these materials during the preparation of circular electrolyte films, the obtained theoretical IL content value should be lower than that of the actual content.

### Materials Characterization

The obtained samples, including electrolyte films and ZIF‐62 crystal/glass, PEO, and LiTFSI materials, were subjected to various characterization methods as described in the following. The powders were pressed into pellets for some measurements using a Graseby Specac press with a 13 mm mold at 5 bar for ≈5 s. XRD patterns were tested by the Empyrean PANalytical diffractometer, with Cu Kα1 radiation (1.5405 Å) in the (2*θ*) range 5°–75° with a step size of 0.013°. The measurement was done at 20 mA and 45 kV. It was noted that the film thickness values of PEO‐LiTFSI (about 90 µm), PEO‐LiTFSI‐1 wt.% glass (about 100 µm), PEO‐LiTFSI‐5 wt.% glass (about 70 µm), PEO‐LiTFSI‐10 wt.% glass (about 150 µm), and PEO‐LiTFSI‐5 wt.% crystal (about 160 µm) for the XRD tests were different.

FTIR spectroscopy measurements were performed using the attenuated total reflectance (ATR) method on a Brüker Lumos FTIR microscope. Morphology analysis was conducted by scanning electron microscopy (SEM, JEM 7001F, JEOL). The used ATR crystal was zinc selenide (ZnSe) and the spectra were collected from 4000 to 600 cm^−1^. ^1^H NMR spectra were measured on a Bruker DRX600 spectrometer with a 5 mm CPP‐TXI probe at 600 MHz. A small amount of ZIF‐62 crystal and glass were prepared in separate NMR tubes. Both were then filled with a solution of DCL (20%)/D_2_O (0.1 mL) and DMSO‐d6 (0.6 mL). Spectral analyses were performed using TopSpin software.

To determine the hardness of the electrolyte films, microindentation experiments were performed using a Nanovea CB500 indenter with a four‐sided diamond Vickers tip (136°). The target load was set to 0.05 N. The internal stepper motor was used to determine the depth. The loading and unloading rates were ≈0.03 and 0.22 N min^−1^, respectively. The measurements were performed under ambient conditions (temperature of ≈22 °C and relative humidity of 35 ± 10%). Films of similar thickness were chosen to ensure results could be compared. Stretching tests were performed to get an insight into the maximum tensile elongation of the different electrolyte films when they were stretched and fractured with the same stretching rate. A ruler was placed as a measuring tool and the film was then stretched slowly alongside the ruler until it fractured.

For the thermal analyses, ZIF‐62 crystals and glasses were analyzed on a Netzssch STA 449 F1 Jupiter instrument. The samples were placed in capsules consisting of an alumina crucible with a lid and heated at a rate of 10 °C min^−1^. Purge argon gas was injected at a rate of 40 mL min^−1^. The thermal properties of the electrolyte films were further analyzed between −60 and 100 °C on an STA 449 F3 Jupiter. For these measurements, the lids on the crucibles were pressed using a mechanical crucible sealing press. The glass transition temperature (*T*_g_) was determined by fitting two linear regression lines and finding their intersection on the heat flow versus temperature plot in the investigated temperature range.

### Electrochemical Measurements

The assembly of 2032 coin‐type cell batteries was performed in a TMAX‐VGB‐6 glovebox. EIS measurements were done on symmetrical SS cells (SS|electrolyte|SS) to determine the ionic conductivity. The experiments were conducted using potentiostatic measurements with a voltage amplitude set to 5 mV and the frequency from 100 mHz to 1 MHz, starting at 1 MHz. Each cell was tested at 30 to 70 °C with a 10 °C interval. The Li^+^ transference number was determined by the Bruce–Vincent method with symmetric lithium metal cells (Li|electrolyte|Li). EIS was conducted in the frequency range from 0.1 Hz to 1 MHz at 60 °C. The electrochemical stability window of the electrolytes was evaluated using LSV with a step size of 1 mV s^−1^. The measurement was performed with Li|electrolyte|SS cells at 60 °C. For full batteries, the cathode electrode was prepared by mixing lithium iron phosphate (LFP, 80 wt.%), Super‐P (10 wt.%), and polyvinylidene fluoride (PVDF, 10 wt.%) with *N*‐methylpyrrolidone (NMP) dispersant. The LFP mass load was about 2 mg cm^−2^. The 1 C rate of full batteries corresponded to 170 mAh g^−1^. For electrochemical tests, the thickness of the used electrolyte films was about 80–100 µm. Galvanostatic cycle tests of full batteries were carried out in the range of 2.5–4 V.

### Molecular Dynamics Simulation of IL on the Surface of ZIF‐62

All the MD simulations were carried out using the Large‐scale Atomic/Molecular Massively Parallel Simulator (LAMMPS) package.^[^ ^42^ ^]^ The interaction between IL (EMIM‐TFSI with 10% LiTFSI) and both ZIF‐62 crystal and glass surfaces was first simulated. The interatomic interactions of the IL molecules were described using the Canongia Lopes & Padua (CL&P) force field, which was one of the most widely used force fields for MD simulations of ILs.^[^ ^43^ ^]^ Since this force field was based on the non‐polarizable method, it can be easily combined with other force fields to simulate the interaction of ILs with other phases. In the present case, the interactions within ZIF‐62 and between ZIF‐62 and IL were simulated using the modified version of the General Amber force field (GAFF), which was used in simulating the absorption behaviors of ZIF materials.^[^ ^44^ ^]^ The point charges of framework atoms were calculated by density functional theory (DFT) simulations using the DDEC method.^[^ ^45^ ^]^ The calculated charges of ZIF‐62 can be found in Figure S24, Supporting Information.

The initial structure of ZIF‐62 crystal and glass was used from the previous work (https://github.com/OxideGlassGroupAAU/DeepZIF (2023)) as the starting structure for creating the surface structure. The unit cell of ZIF‐62 crystal (consisting of 263 atoms) was replicated to 15 × 15 × 2, which yielded a system size of around 235 × 235 × 36 Å^3^. Meanwhile, the ZIF‐62 glass (consisting of 3996 atoms) was replicated to 6 × 6 × 1, yielding a system size of around 222 × 222 × 37 Å^3^. Then, both the upper and lower parts of ZIF‐62 crystal and glass were cleaved to obtain the surface structures. To ensure the stability of the models, incomplete linkers induced by the cleavage were removed. The thickness of the remaining slabs was around 12 Å. Then, a pre‐equilibrated IL droplet consisting of 1000 EMIM‐TFSI molecules was placed near the surface of the ZIF‐62 crystal and glass substrates at a distance of around 5 Å. The combined systems were first dynamically equilibrated in the *NVT* ensemble at 300 K for 6 ns to ensure the full contact of the IL with the substrates. The systems were then further equilibrated under 300, 400, 450, 500, 550, and 600 K for another 6 ns to reach an equilibrated state. The ZIF‐62 substrates were kept fixed during the whole simulation. The final structures under different temperatures were used for contact angle and absorption energy calculations. The contact angle was calculated using the method proposed in ref. [46], while the interfacial adsorption energy was calculated as

(1) $$E_{\mathrm{adsorption}}=E_{\mathrm{total}}-E_{\mathrm{IL}}-E_{\mathrm{ZIF}}$$

where *E*_total_ is the total potential energy of the combined system, *E*_IL_ is the potential energy of IL, and *E*_ZIF_ is the potential energy of the ZIF‐62 substrate. The calculated *E*_adsorption_ was then normalized by the number of atoms within IL molecules (33 300 in this case). The atomic volume of Li^+^ was calculated from Voronoi tessellation. The MSD of Li^+^ within IL on different ZIF‐62 surfaces during the equilibration process was calculated as

(2) $$\mathrm{MSD}\;\left(t\right)=\frac{1}{N}\;\sum_{i}^{N}{\left[r_{i}\left(t\right)-r_{i}\left(0\right)\right]}^{2}$$

### Simulated Diffusion Behavior of Li^+^ in IL

Based on the Young–Laplace theory,^[^ ^36^ ^]^ the different wettability of IL led to different states of confinement applied to IL on the crystalline and glassy ZIF‐62 surfaces. Therefore, the diffusion behaviors of Li^+^ in the IL under different pressures were simulated. To this end, an IL system was first built with 10% EMIM‐TFSI replaced by Li‐TFSI, consisting of 900 EMIM‐TFSI and 100 Li‐TFSI molecules. The systems were then equilibrated in the *NPT* ensemble at 300 K and different pressures (0, 0.5, and 1 GPa) for 250 ps to converge the density. The resulting configurations were used for the MSD calculations (Equation (2)) in the temperature range of 600–800 K with an interval of 50 K; the diffusion coefficient *D* of Li ions was calculated from the MSD curves (Equation (3)); and the temperature dependence of *D* was fitted by the Arrhenius equation (Equation (4)) to obtain the activation energy and the room temperature diffusivity

(3) $$D=\frac{1}{6}\;\lim_{t\to \infty }\frac{d\langle \mathrm{MSD}\rangle }{dt}$$

(4) $$D=D_{0}\;\exp\left(\frac{-E_{a}}{k_{B}T}\right)$$

where *N* is the total number of Li ions, **r** *_i_* is the position of *i*_th_ Li^+^, *t* is the simulation time, *k*_B_ is the Boltzmann constant, and *T* is the temperature.

## Conflict of Interest

The authors declare no conflict of interest.

## Supporting information

Supporting Information

## Data Availability

The data that support the findings of this study are available from the corresponding author upon reasonable request.
