# Supplementary material for: Metal‐Organic Framework Glass as a Functional Filler Enables Enhanced Performance of Solid‐State Polymer Electrolytes for Lithium Metal Batteries
Source: Adv Sci (Weinh). 2023 Dec 25;11(10):2306698. doi: 10.1002/advs.202306698 (PMC10933666; doi:10.1002/advs.202306698)
Supplement: Supplementary file 1 — Supporting Information [file ADVS-11-2306698-s001.pdf]

ADVANCED SCIENCE
Open Access

Supporting Information

for *Adv. Sci.*, DOI 10.1002/advs.202306698

Metal-Organic Framework Glass as a Functional Filler Enables Enhanced Performance of Solid-State Polymer Electrolytes for Lithium Metal Batteries

*Junwei Ding, Tao Du, Emil H. Thomsen, David Andresen, Mathias R. Fischer, Anders K. Møller, Andreas R. Petersen, Andreas K. Pedersen, Lars R. Jensen, Shiwen Wang and Morten M. Smedskjaer**

Metal-Organic Framework Glass as a Functional Filler Enables Enhanced Performance of Solid-State Polymer Electrolytes for Lithium Metal Batteries

Junwei Ding[a,1], Tao Du[a,1], Emil H. Thomsen[a], David Andresen[a], Mathias R. Fischer[a], Anders K. Møller[a], Andreas R. Petersen[a], Andreas K. Pedersen[a], Lars R. Jensen[b], Shiwen Wang[c], Morten M. Smedskjaer[a,*]

[a]Department of Chemistry and Bioscience, Aalborg University, Aalborg, Denmark.

[b]Department of Materials and Production, Aalborg University, Aalborg, Denmark.

[c]College of New Energy, Zhengzhou University of Light Industry, Zhengzhou 450002, China.

*Corresponding author. Email: mos@bio.aau.dk

[1]These authors contributed equally to this work.

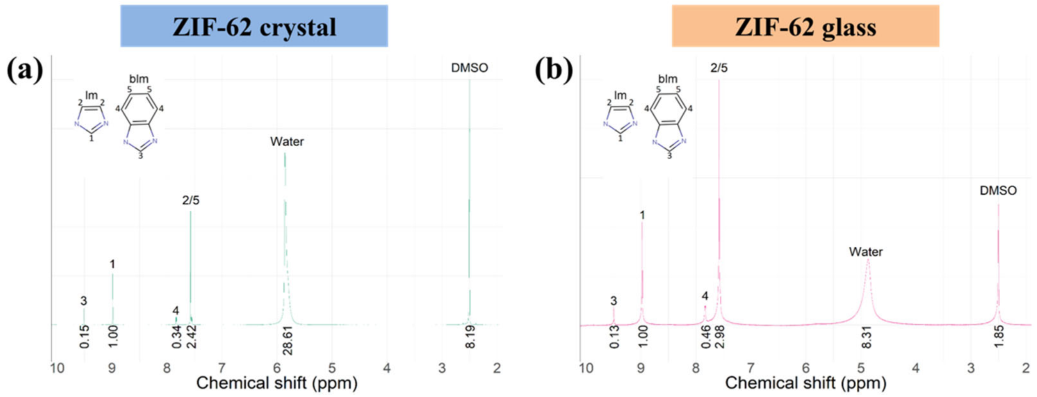

Figure S1. 1H NMR spectra of (a) ZIF-62 crystal and (b) ZIF-62 glass. The area of the peaks can be seen below each peak. The given number for the corresponding H-atom in chemical structural formula of benzimidazole and imidazole is displayed. From the area under the peak at 9 and 9.5 ppm, the benzimidazole/imidazole ratio can be determined. The ratio is then used to find the composition when assuming two linkers per zinc atom and no free linkers, which yields; $ZnIm_{1.74}bIm_{0.26}$ and $ZnIm_{1.77}bIm_{0.23}$ for crystal and glass, respectively. Interestingly, the water peak shifts between the two measurements. This may be caused by differences in solution concentration and/or pH change. However, this is likely of little to no consequence for the determination of the chemical composition of the ZIF-62.

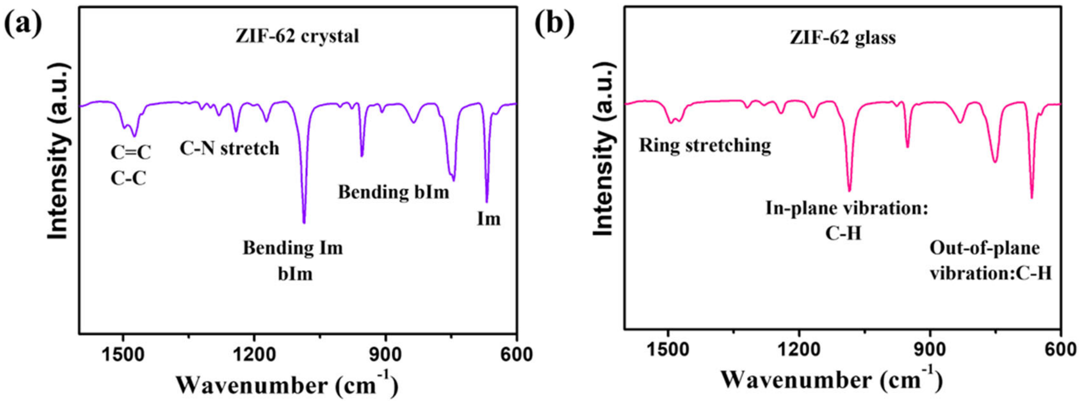

Figure S2. (a, b) FTIR spectra of the ZIF-62 crystal and ZIF-62 glass, respectively.

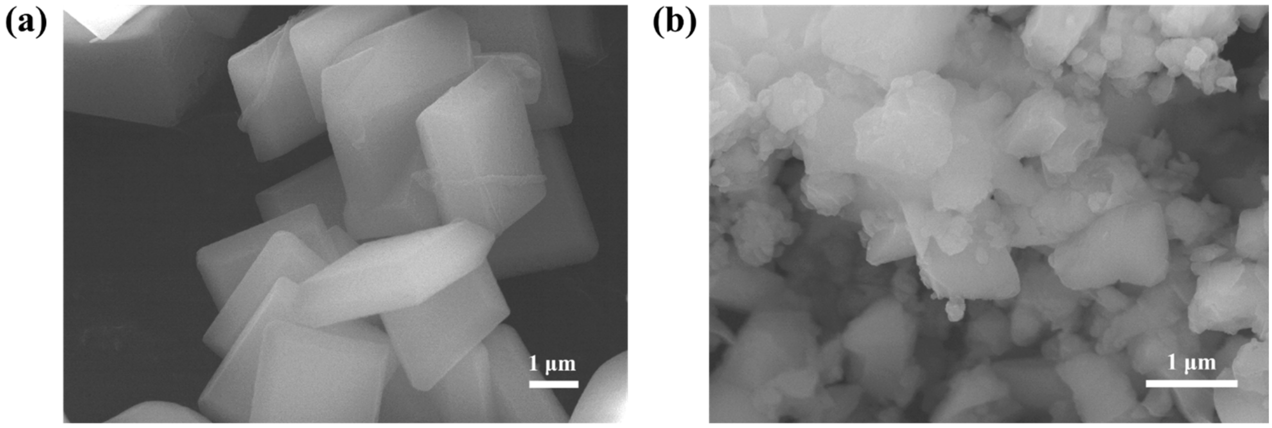

Figure S3. Scanning electron microscopy images of the (a) ZIF-62 crystal and (b) ZIF-62 glass samples. During the melt-quenching process of ZIF-62 crystal precursor, the molten ZIF-62 has a very high viscosity, resulting in very limited fluidity. Therefore, the surface morphology of the ZIF-62 glass particle is partially inherited from the ZIF-62 crystal particle precursor, as also observed elsewhere.[1]

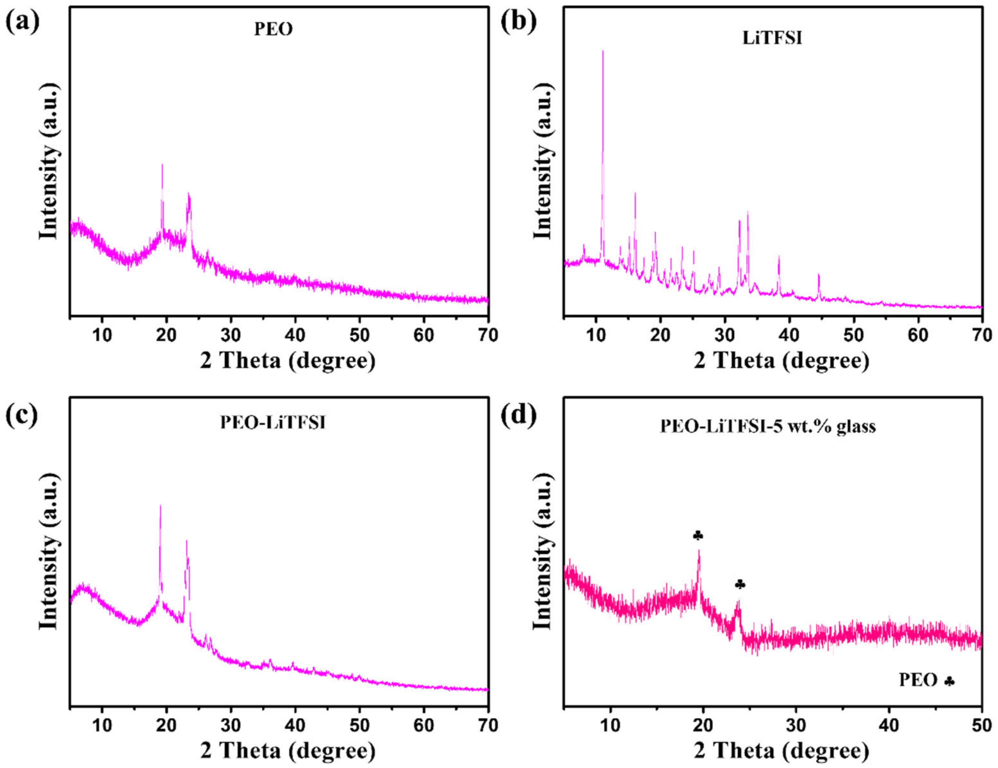

Figure S4. XRD patterns of the (a) pure PEO film, (b) LiTFSI powder, (c) PEO-LiTFSI film, and (d) PEO-LiTFSI-5 wt.% glass electrolyte film.

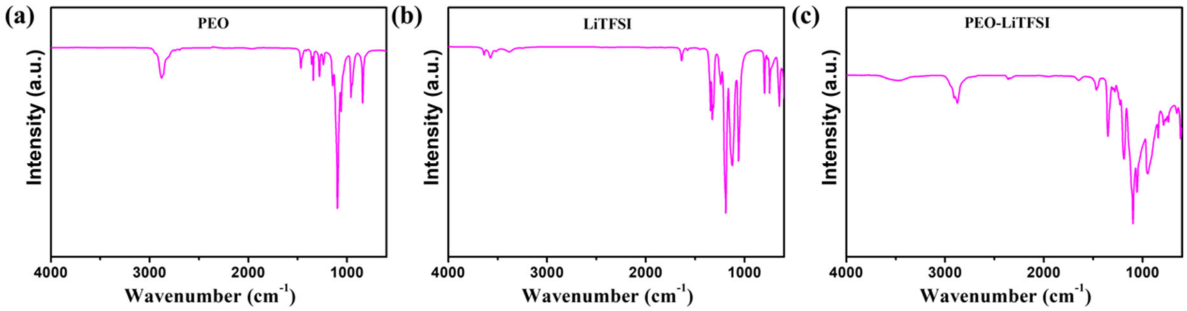

Figure S5. FTIR spectra of the (a) pure PEO film, (a) LiTFSI powder, and (a) PEO-LiTFSI film.

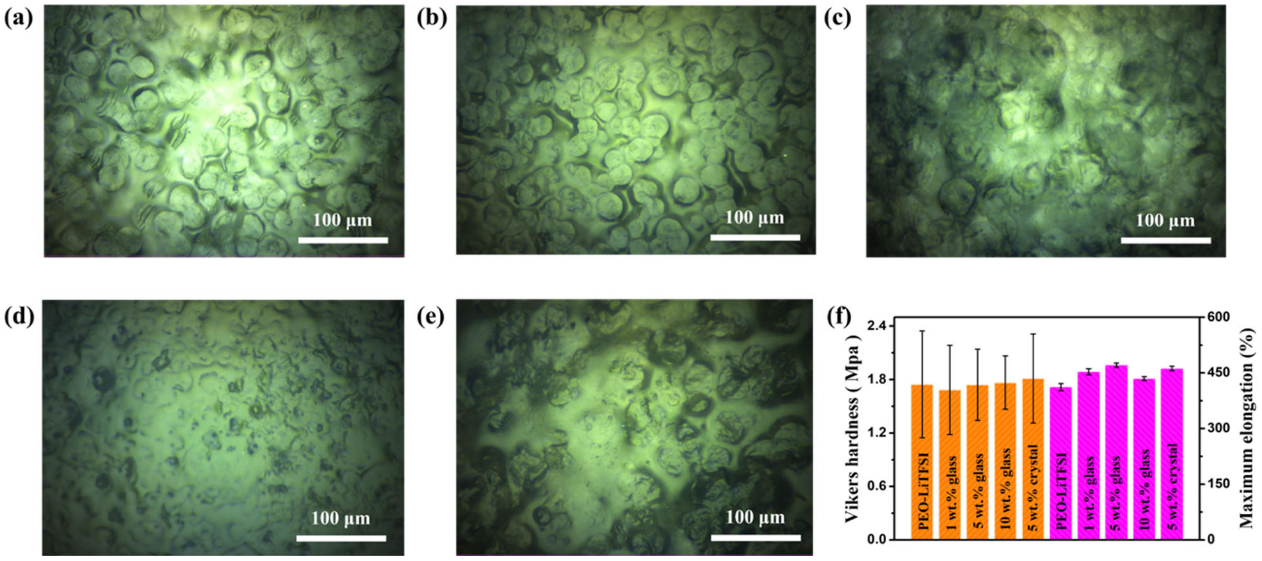

Figure S6. (a-e) Optical microscope images of (a) PEO-LiTFSI, (b) PEO-LiTFSI-1 wt.% glass, (c) PEO-LiTFSI-5 wt.% glass, (d) PEO-LiTFSI-10 wt.% glass, and (e) PEO-LiTFSI-5 wt.% crystal electrolytes. (f) Vikers hardness (orange) and maximum elongation (magenta) comparsion of five electrolytes.

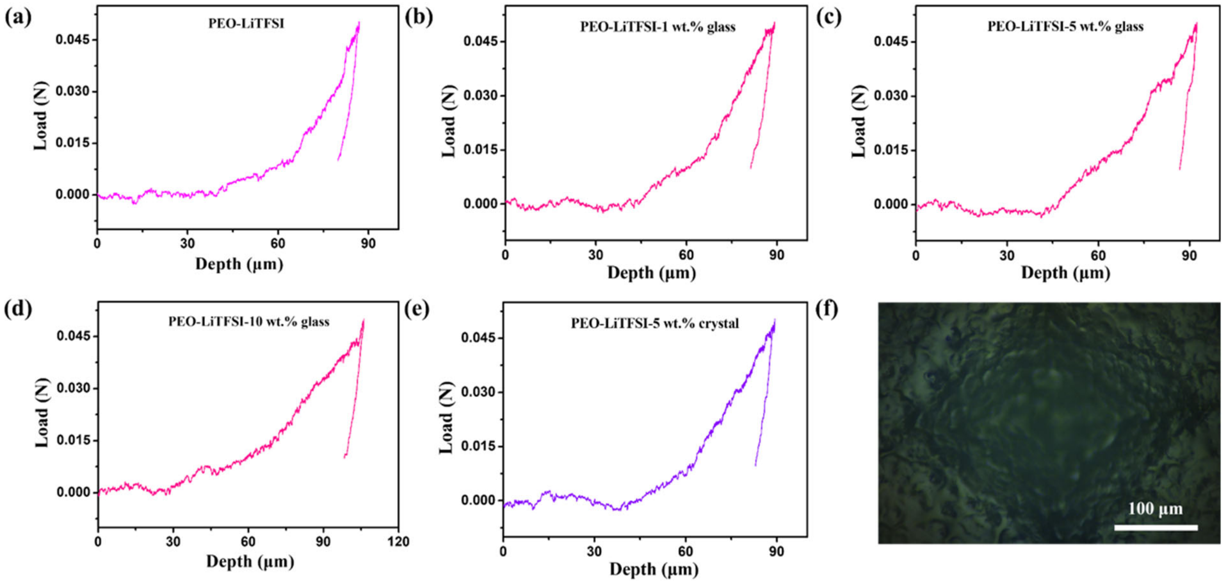

Figure S7. Loading/unloading curves of (a) PEO-LiTFSI, (b) PEO-LiTFSI-1 wt.% glass, (c) PEO-LiTFSI-5 wt.% glass, (d) PEO-LiTFSI-10 wt.% glass, and (e) PEO-LiTFSI-5 wt.% crystal electrolyte films. (f) Optical microscopy indentation image. Notably, due to the large surface roughness of the PEO-based electrolyte film, it is difficult for the indenter tip to locate the sample surface at the beginning of loading process, which is consistent with previous findings.[2] However, the values of hardness and modulus are calculated from the unloading curve, i.e., the hardness is determined by fitting the first one third of the unloading curve to a linear model. During the indentation tests, we observed some differences in the hardness and reduced modulus values obtained at different loading/unloading rates, indicating that the prepared electrolyte film has the time-dependent mechanical behavior, which is consistent with previously reportes.[3]

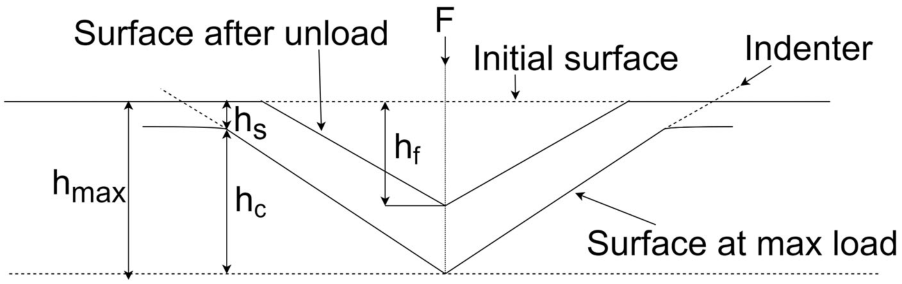

Figure S8. Schematic of the relevant parameters during indentation test. The stiffness, S, of the contact between the indenter and the film is determined as the slope, dP/dh, at the initial unload (where the depth h is equal to the maximum depth, h_{max}). The stiffness is determined by fitting the first 1/3 of the unloading curve to a linear model.

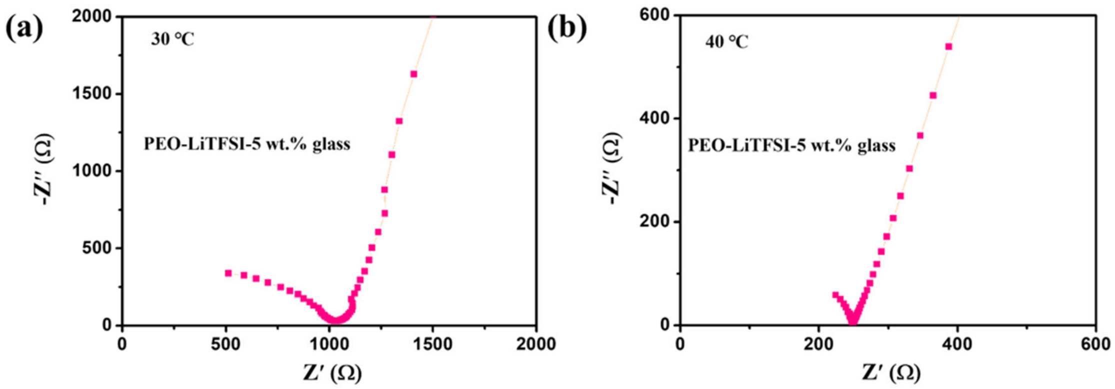

Figure S9. Electrochemical impedance spectroscopy (EIS) curves of the PEO-LiTFSI-5 wt.% glass electrolytes based Li-Li symmetric batteries at (a) 30 °C and (b) 40 °C.

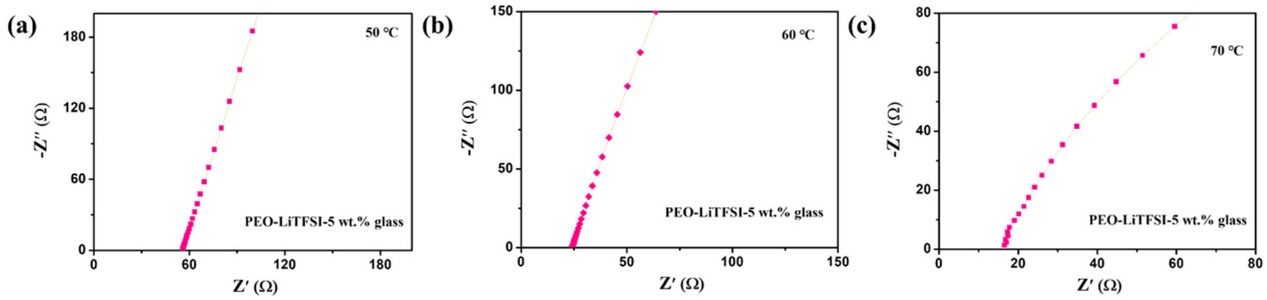

Figure S10. EIS curves of the PEO-LiTFSI-5 wt.% glass electrolytes based Li-Li symmetric batteries at (a) 50 °C , (a) 60 °C, and (b) 70 °C.

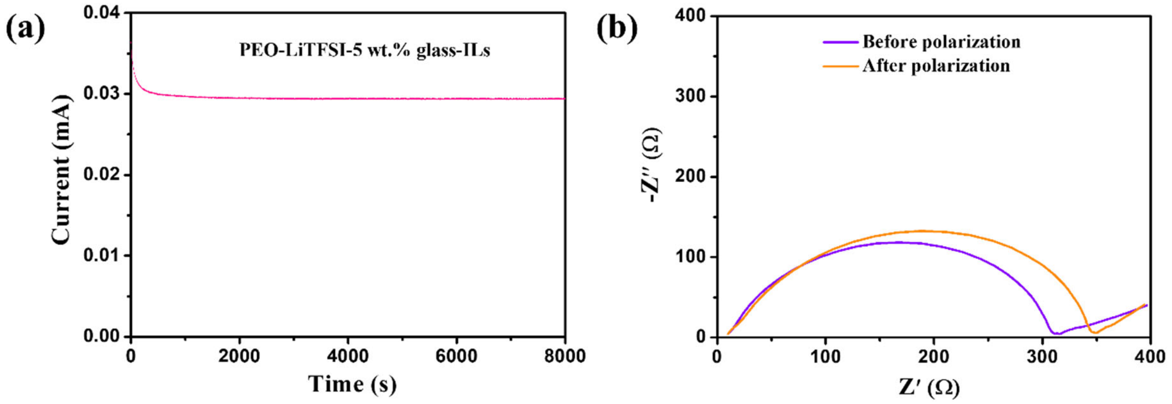

Figure S11. (a) Chronoamperometric curve of the PEO-LiTFSI-5 wt.% glass-ILs electrolyte based Li-Li symmetric batteries at 60 °C. (b) Impedance curves before and after polarization at 60 °C.

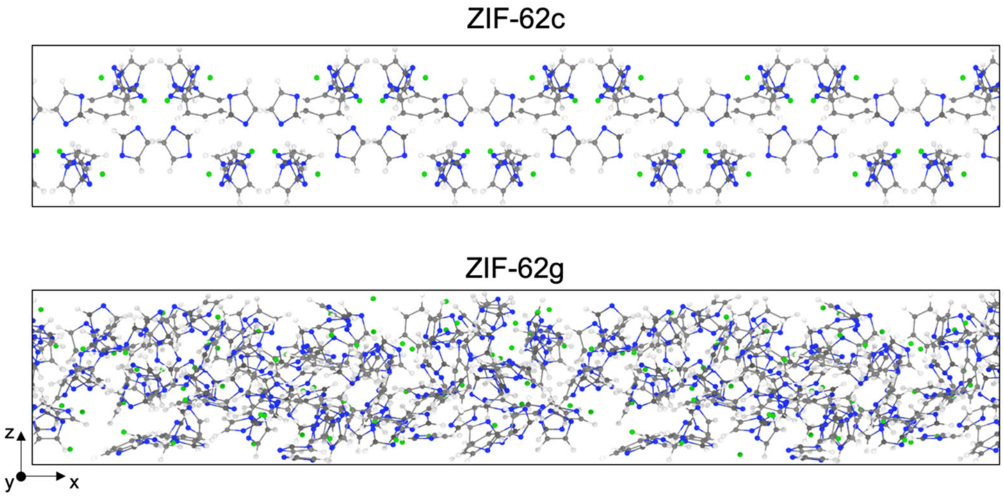

Figure S12. Schematic structures of ZIF-62 crystal (top) and ZIF-62 glass (bottom) used for the MD simulations.

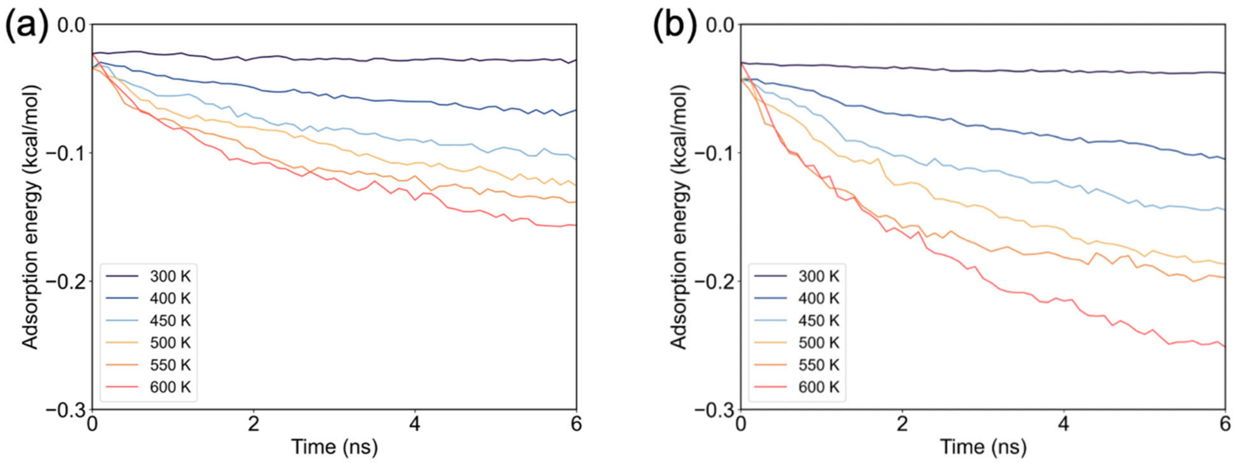

Figure S13. Evolution of absorption energy of ILs on the surface of (a) ZIF-62 crystal and (b) ZIF-62 glass as a function of temperature.

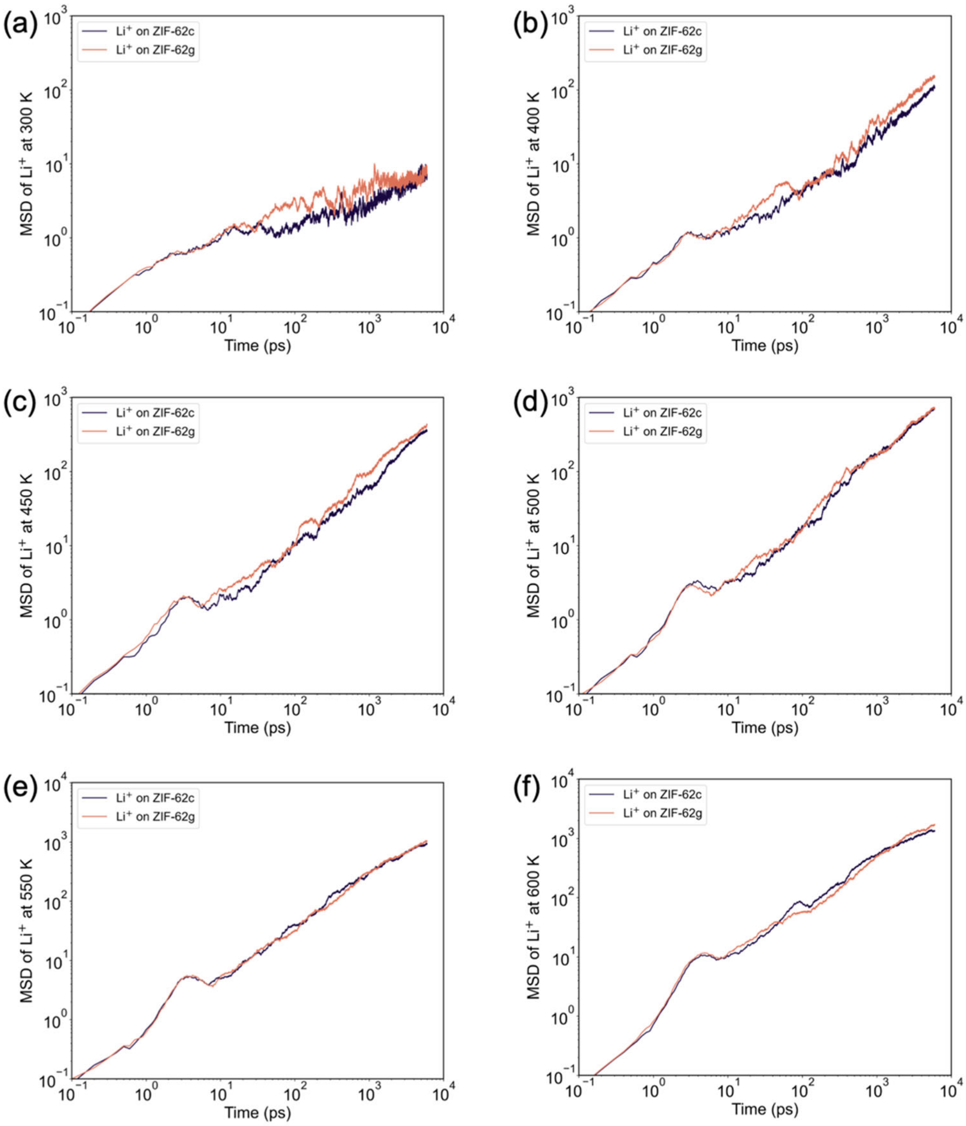

Figure S14. Simulated MSD curves of Li^+ in the ionic liquid on different ZIF-62 substrates at (a) 300 K, (b) 400 K, (c) 450 K, (d) 500 K, (e) 550 K, and (f) 600 K.

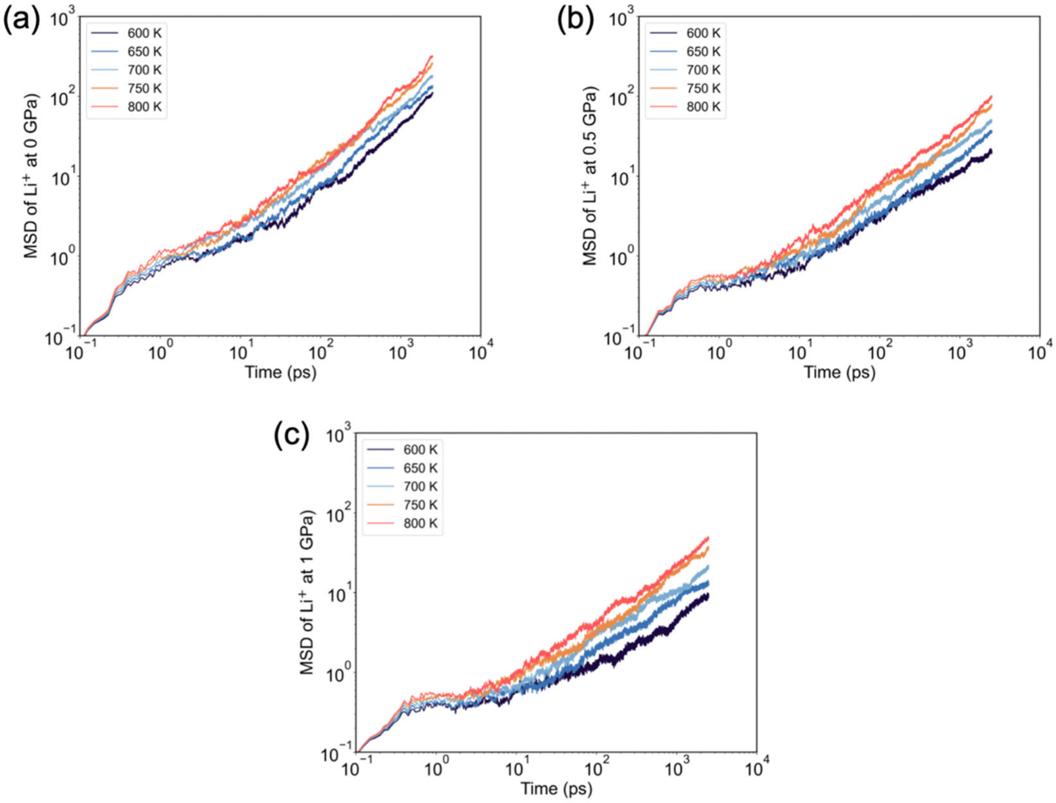

Figure S15. Simulated MSD curves of Li^+ in the ionic liquid at different temperatures when subjected to a pressure of (a) 0 GPa, (b) 0.5 GPa, and (c) 1 GPa.

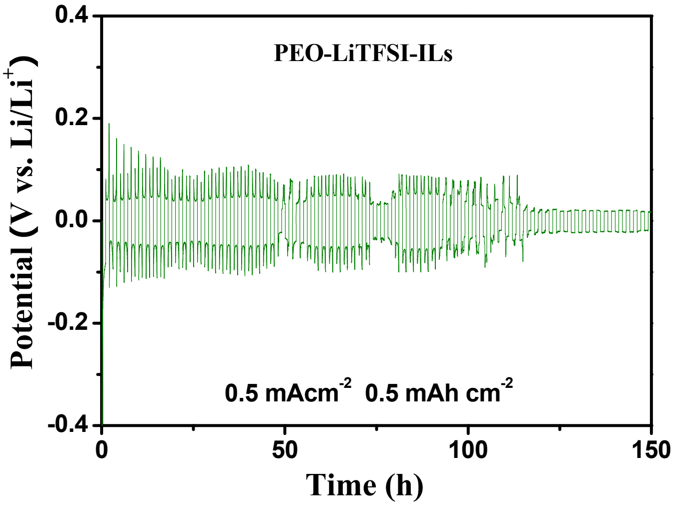

Figure S16. Voltage evolution curve of the PEO-LiTFSI-ILs electrolyte based Li-Li symmetric batteries at 60 °C.

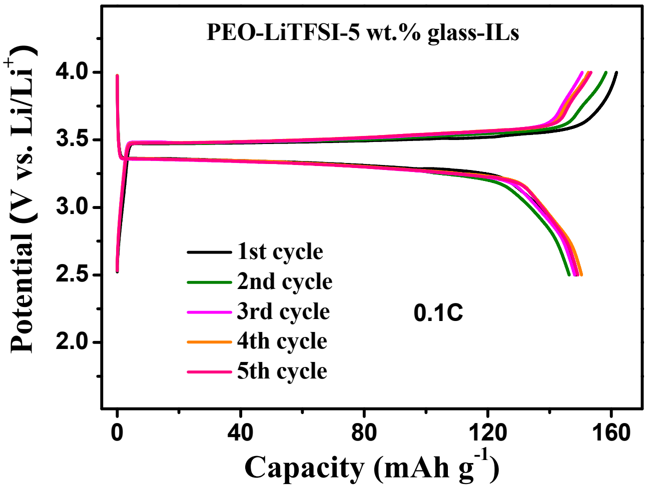

Figure S17. Voltage-capacity curves of the PEO-LiTFSI-5 wt.% glass-ILs electrolyte based Li-LFP full batteries at 60 °C at 0.1 C.

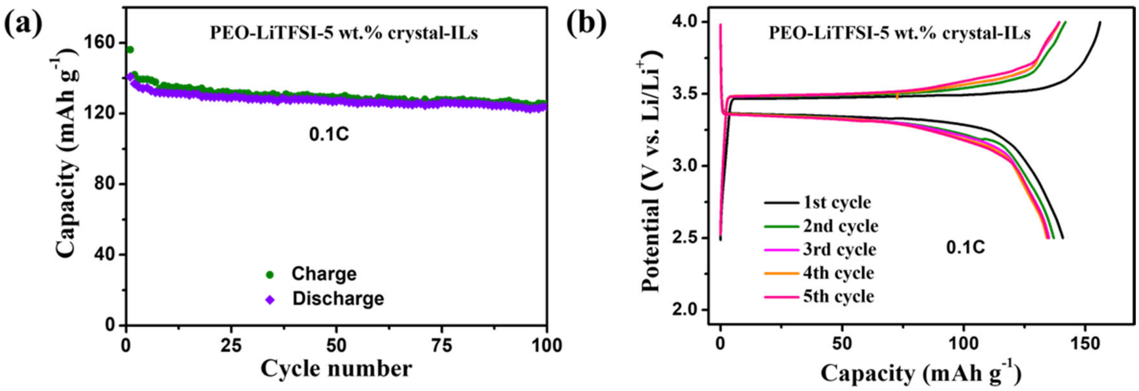

Figure S18. (a) Cyclic performance and (b) the corresponding voltage-capacity curves of the PEO-LiTFSI-5 wt.% crystal-ILs electrolyte based Li-LFP full batteries at 60 °C.

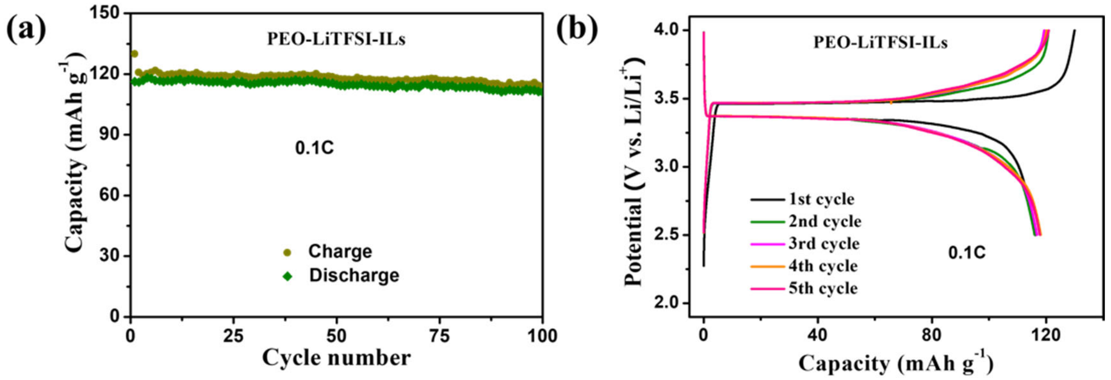

Figure S19. (a) Cyclic performance and (b) the corresponding voltage-capacity curves of the PEO-LiTFSI-ILs electrolyte based Li-LFP full batteries at 60 °C.

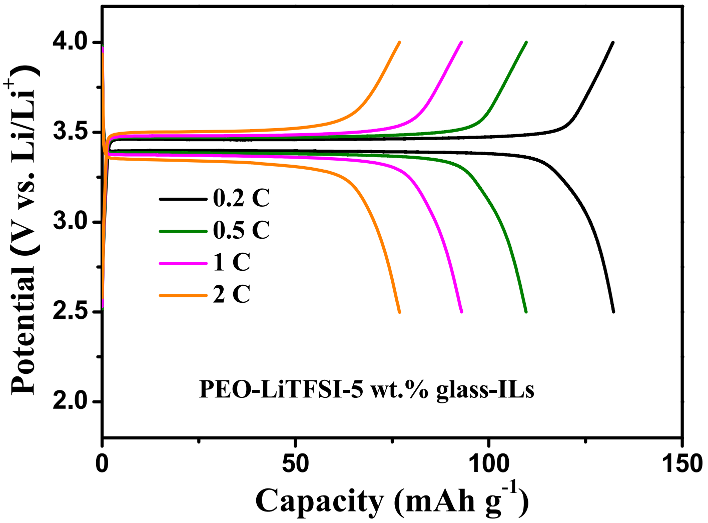

Figure S20. Voltage-capacity curves with different current densities of the PEO-LiTFSI-5 wt.% glass-ILs electrolyte based Li-LFP full batteries at 60 °C.

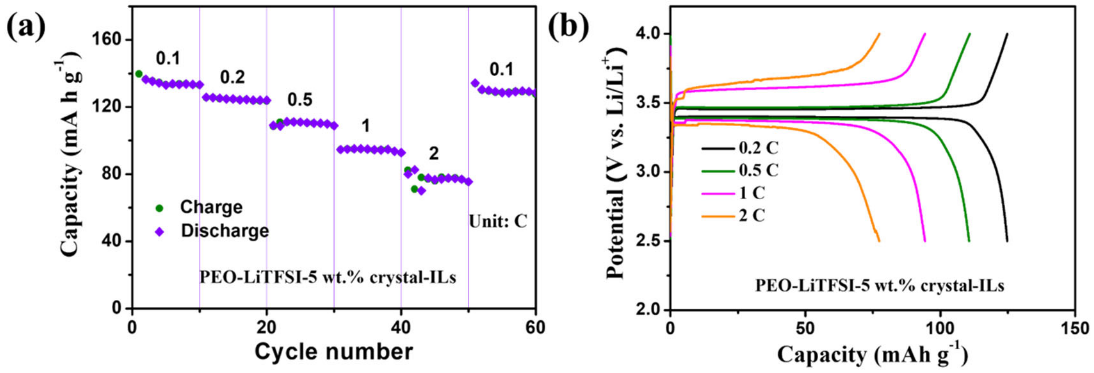

Figure S21. (a) Rate performance and (b) corresponding voltage-capacity curves of the PEO-LiTFSI-5 wt.% crystal-ILs electrolyte based Li-LFP full batteries at 60 °C.

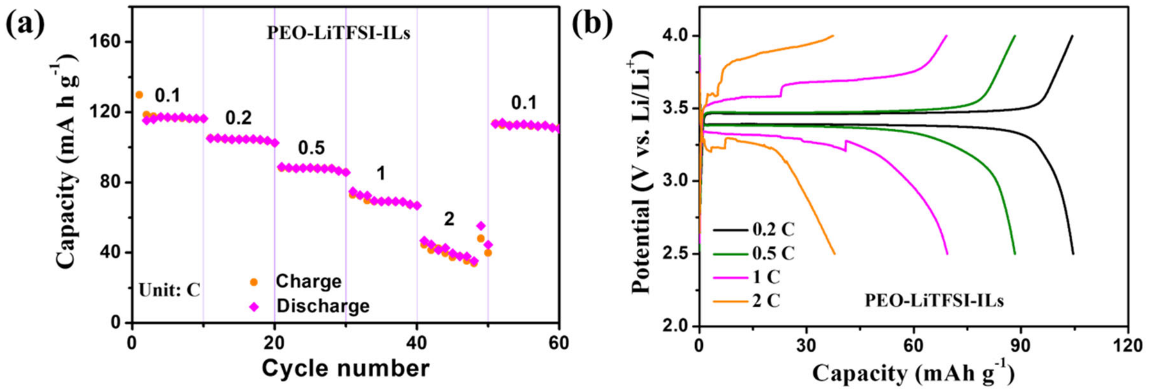

Figure S22. (a) Rate performance and (b) corresponding voltage-capacity curves of the PEO-LiTFSI-ILs electrolyte based Li-LFP full batteries at 60 °C.

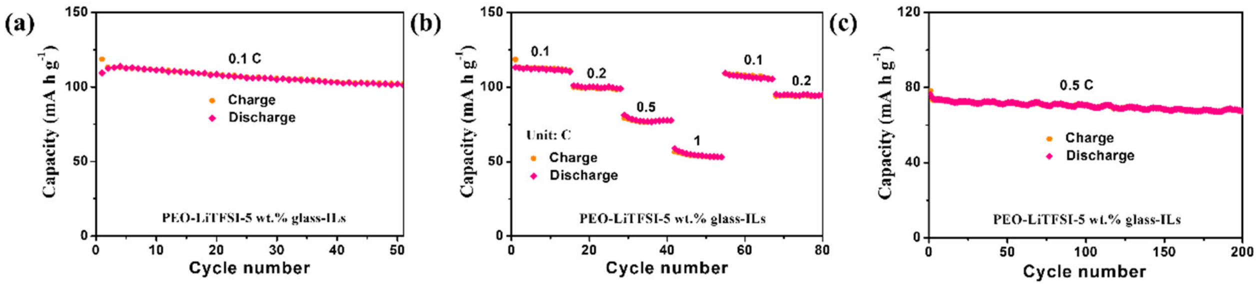

Figure S23. (a) Cycle performance, (b) rate performance, and (c) long cycle stability of the PEO-LiTFSI-5 wt.% glass-IL electrolyte based Li-LFP full batteries at room temperatrue (25 °C).

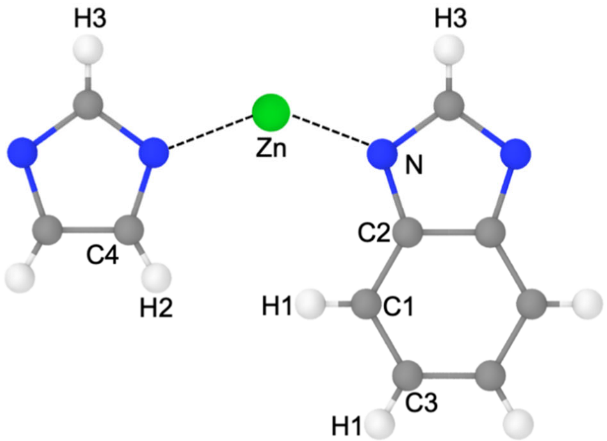

Type	q(e)
C1	-0.1496
C2	0.1342
C3	-0.0786
C4	0.1283
H1	0.1114
H2	0.1022
H3	0.0708
N	-0.2756
Zn	0.5927

Figure S24. Atom types in ZIF-62 and calculated charges from DFT using the DDEC method.

Table S1. Hardness and modulus values obtained from indentation measurements.

Electrolyte film	Vickers hardness (MPa)	Reduced modulus (MPa)
PEO-LiTFSI	1.8 ± 0.6	29 ± 8
PEO-LiTFSI-1 wt.% glass	1.7 ± 0.5	31 ± 6
PEO-LiTFSI-5 wt.% glass	1.7 ± 0.4	30 ± 5
PEO-LiTFSI-10 wt.% glass	1.8 ± 0.3	29 ± 6
PEO-LiTFSI-5 wt.% crystal	1.8 ± 0.5	31 ± 7

Table S2. Comparison of the performance of solid-state electrolytes.

Type	Ionic conductivity ($S\ cm^{-1}$)	Ion transference number	Stability (V)	Symmetrical battery	Ref.
HKUST-1 crystal	3.5×10^{-4} (50 °C)	0.38	4.71	240 ($0.1\ mA\ cm^{-2}$)	[4]
ZIF-90 crystal	7.2×10^{-5} (20 °C)	0.44	4.8	700 ($0.1\ mA\ cm^{-2}$)	[5]
UiO-66 crystal	6.3×10^{-4} (60 °C)	0.72	4.97	200 ($0.5\ mA\ cm^{-2}$)	[6]
Li_2OHBr	4.5×10^{-4} (60 °C)	0.33	NA	200 ($0.1\ mA\ cm^{-2}$)	[7]
ZIF-4 glass	1.61×10^{-4} (30 °C)	0.885	4.0	400 ($0.1\ mA\ cm^{-2}$)	[8]
UiO-66 crystal	4.56×10^{-4} (60 °C)	0.36	5.0	350 ($0.1\ mA\ cm^{-2}$)	[9]
Covalent organic polymer	1.2×10^{-3} (80 °C)	0.28	4.7	600 ($0.1\ mA\ cm^{-2}$)	[10]
Boron nitride	4.5×10^{-4} (60 °C)	0.54	4.71	180 ($0.02\ mA\ cm^{-2}$)	[11]
$Li_{0.33}La_{0.55}TiO_3$	1.2×10^{-3} (60 °C)	0.48	4.7	300 ($0.15\ mA\ cm^{-2}$)	[12]
**ZIF-62 glass**	**2.41×10^{-4} (30 °C)**	**0.39**	**4.7**	**300 ($0.5\ mA\ cm^{-2}$)**	**This work**

Supporting References

[1] V. Nozari, C. Calahoo, L. Longley, T. D. Bennett, L. Wondraczek, J. Chem. Phys. **2020**, 153, 204501.

[2] D. Pérez-Calixto, S. Amat-Shapiro, D. Zamarrón-Hernández, G. Vázquez-Victorio, P. H. Puech, M. Hautefeuille, *Polymers* **2021**, 13, 629.

[3] a) J. Minster, O. Blahova, J. Lukes, J. Nemecek, *Mech.Time Depend. Mater.* **2010**, 14, 243; b) W. Qin, Q. Yang, Y. He, L. Zhang, H. Hu, *Mech. Mater.* **2023**, 185, 104773.

[4] C. C. Sun, A. Yusuf, S. W. Li, X. L. Qi, Y. Ma, D. Y. Wang, *Chem. Eng. J.* **2021**, 414, 128702.

[5] Z. Lei, J. Shen, J. Wang, Q. Qiu, G. Zhang, S. S. Chi, H. Xu, S. Li, W. Zhang, Y. Zhao, Y. Deng, C. Wang, *Chem. Eng. J.* **2021**, 412, 128733.

[6] H. Huo, B. Wu, T. Zhang, X. Zheng, L. Ge, T. Xu, X. Guo, X. Sun, *Energy Storage Mater.* **2019**, 18, 59.

[7] Y. Ye, Z. Deng, L. Gao, K. Niu, R. Zhao, J. Bian, S. Li, H. Lin, J. Zhu, Y. Zhao, *ACS Appl. Mater. Interfaces* **2021**, 13, 28108.

[8] G. Jiang, C. Qu, F. Xu, E. Zhang, Q. Lu, X. Cai, S. Hausdorf, H. Wang, S. Kaskel, *Adv. Funct. Mater.* **2021**, 31, 2104300.

[9] G. Lu, H. Wei, C. Shen, F. Zhou, M. Zhang, Y. Chen, H. Jin, J. Li, G. Chen, J. Wang, S. Wang, *ACS Appl. Mater. Interfaces* **2022**, 14, 45476.

[10] Y. Wang, H. Ji, X. Zhang, J. Shi, X. Li, X. Jiang, X. Qu, *ACS Appl. Mater. Interfaces* **2021**, 13, 16469.

[11] X. Zhang, W. Guo, L. Zhou, Q. Xu, Y. Min, *J. Mater. Chem. A* **2021**, 9, 20530.

[12] K. Liu, R. Zhang, J. Sun, M. Wu, T. Zhao, *ACS Appl. Mater. Interfaces* **2019**, 11, 46930.
